# Almost periodic synchronization of quaternion-valued shunting inhibitory cellular neural networks with mixed delays via state-feedback control

**DOI:** 10.1371/journal.pone.0198297

**Published:** 2018-06-07

**Authors:** Yongkun Li, Huimei Wang

**Affiliations:** 1 Department of Mathematics, Yunnan University, Kunming, Yunnan 650091, China; 2 Department of Mathematics, Kunming University, Kunming, Yunnan 650214, China; Northwestern University, UNITED STATES

## Abstract

This paper studies the drive-response synchronization for quaternion-valued shunting inhibitory cellular neural networks (QVSICNNs) with mixed delays. First, QVSICNN is decomposed into an equivalent real-valued system in order to avoid the non-commutativity of the multiplicity. Then, the existence of almost periodic solutions is obtained based on the Banach fixed point theorem. An novel state-feedback controller is designed to ensure the global exponential almost periodic synchronization. At the end of the paper, an example is given to illustrate the effectiveness of the obtained results.

## Introduction

Quaternion was first proposed by Hamilton [1] in 1853. However, because of the non-commutativity of quaternion multiplicity, the development on quaternion was quite slow. Fortunately, with the development of modern science, the quaternion has been widely used in attitude control, quantum mechanics, computer graphics and so on, see [2–5] and references therein. In recent years, quaternion has attracted scholars from many fields, especially, the scholars in the field of neural network research. The quaternion-valued neural networks (QVNNs), as an special case of Clifford-valued neural networks [6], can be thought of as an extension of complex-valued neural networks (CVNNs) and real-valued neural networks (RVNNs). In fact, QVNNs can be applied to engineering and science. A great deal of studies have shown that, for the three dimensional data including color images and body images, via direct coding, QVNNs can do the process with high-efficiency [7]. Indeed, based on the three primary colors and Hamilton rules of quaternion, one can realize the color face recognition efficiently. The quaternion representation treats the color image and dictionary in a holistic manner, while the real representation can only treat the three colors channels separately [8, 9]. Since all of these applications strongly rely on the dynamics of QVNNs, many researchers have studied some dynamical behaviours of QVNNs ([10–15]) recently.

On the one hand, after Bouzerdount and Pinter’s [16] new class of cellular neural networks, namely the shunting inhibitory cellular neural networks (SICNNs), many studies have been focusing on the SICNNs, especially about the dynamical behaviors. Because of the wide applications of SICNNs in psychophysics [17], speech [18], perception [19], robotics [20], adaptive pattern recognition [21, 22], vision [23, 24], and image processing [25], moreover, time delays are unavoidable in a realistic system, there have been extensive results about the sufficient conditions on the problem of the existence and stability of equilibrium, periodic, anti-periodic solutions of SICNNs with time delays, see [26–29] and references therein. Besides, it is well known that the almost periodic phenomenon is more universal than the periodic one in real world. In the past few years, many researchers devoted to study the almost periodic problem of SICNNs with time delays ([30–37]).

On the other hand, synchronization is a very common phenomenon in real systems, which indicates that two or more systems adjust each other to lead to a common dynamical behavior. By synchronization, we can understand an unknown system from the well-known systems. Pecora and Carroll [38] proposed a method to synchronize two identical chaotic systems with different initial values in 1990, from then on, the problem of synchronization has attracted scholars from various fields such as information science [39, 40], secure communication [41, 42] and chemical reactions [43, 44]. In particular, in the field of neural networks, much attention has been focusing on this topic, see [45–54] and references therein. At present, there are some results about the synchronization for complex-valued neural networks [55–58]. However, as far as we know, till now there is still no result about the almost periodic synchronization of SICNNs, not to speak of QVSICNNs.

In this paper, we study the QVSICNNs with time varying and distributed delays.

The paper is organized as follows. In Section 2, some preliminaries and notations are introduced. In Section 3, the sufficient conditions for the existence of almost periodic solutions of system (1) are obtained. In Section 4, the global exponential synchronization is studied. In Section 5, the effectiveness and feasibility of the proposed methods in this paper are shown by a numerical example.

## Problem description and preliminaries

We denote the skew field of quaternion by

$$\begin{array}{c} Q:=\{x=x^{R}+ix^{I}++jx^{J}+kx^{K}\}, \end{array}$$

where *x*^*R*^, *x*^*I*^, *x*^*J*^, *x*^*K*^ are real numbers and the elements *i*, *j*, *k* obey the Hamilton’s multiplication rules:

$$\begin{array}{c} ij=-ji=k,\;\;jk=-kj=i,\;\;ki=-ik=j,\;\;i^{2}=j^{2}=k^{2}=ijk=-1. \end{array}$$

In this paper, the model of the shunting inhibitory cellular neural networks with mixed time delays is defined as follows:

$$\begin{array}{ccc} x_{pq}^{\prime }\left(t\right) & = & -a_{pq}\left(t\right)x_{pq}\left(t\right)-\sum_{C_{kl}\in N_{r}\left(p,q\right)}B_{pq}^{kl}\left(t\right)f\left(x_{kl}\left(t\right)\right)x_{pq}\left(t\right)\\ & & -\sum_{C_{kl}\in N_{s}\left(p,q\right)}C_{pq}^{kl}\left(t\right)g\left(x_{kl}\left(t-\tau \left(t\right)\right)\right)x_{pq}\left(t\right)\\ & & -\sum_{C_{kl}\in N_{u}\left(p,q\right)}D_{pq}^{kl}\left(t\right)\int_{0}^{+\infty }K_{pq}\left(u\right)h\left(x_{kl}\left(t-u\right)\right)\text{d}ux_{pq}\left(t\right)+T_{pq}\left(t\right), \end{array}$$ (1)

where 1 ≤ *p* ≤ *m*, 1 ≤ *q* ≤ *n*, for the convenience, we denote pq∈{11,12,…,1n,…,m1,m2,…,mn}:=J; *C*_*pq*_ denotes the cell at the position (*p*, *q*) of the lattice; the *r*-neighborhood of *C*_*pq*_ is defined as

$$\begin{array}{c} N_{r}\left(p,q\right)=\{C_{kl}:\max(|k-p|,|l-q|)\leq r,pq\in J\}, \end{array}$$

and *N*_*s*_(*p*, *q*), *N*_*u*_(*p*, *q*) are similarly specified; $x_{pq}\in Q$ is the activity of the cell *C*_*pq*_, $T_{pq}:Q\to Q$ is the external input to *C*_*pq*_, *a*_*pq*_(*t*)>0 represents the passive decay rate of the cell activity; $B_{pq}^{kl}\left(t\right),C_{pq}^{kl}\left(t\right),D_{pq}^{kl}\left(t\right)\geq 0$ are the connection or coupling strength of postsynaptic activity of the cell transmitted to *C*_*pq*_, and the activity functions f,g,h:Q→Q are the continuous functions representing the output or firing rate of the cell *C*_*pq*_; *τ*(*t*) ≥ 0 denotes the transmission time varying delay; *K*_*pq*_(*t*) denotes the transmission delay kernels.

The initial conditions associated with system (1) are of the form

$$\begin{array}{c} x_{pq}\left(s\right)=\varphi_{pq}\left(s\right),\;s\in \left(-\infty ,0\right],\;\;pq\in J, \end{array}$$

where $\varphi_{pq}\left(s\right)=\varphi_{pq}^{R}\left(s\right)+i\varphi_{pq}^{I}\left(s\right)+j\varphi_{pq}^{J}\left(s\right)+k\varphi_{pq}^{K}\left(s\right),\varphi_{pq}^{K},\varphi_{pq}^{I},\varphi_{pq}^{J},\varphi_{pq}^{K}:\left(-\infty ,0\right]\to R$ are bounded continuous functions.

Now, we introduce some relevant definitions and basic lemmas.

**Definition 1**. [59] *A function* $x\in C(R,R^{n})$ *is said to be almost periodic if, for any* *ϵ* > 0, *it is possible to find a real number* *l* = *l*(*ϵ*) > 0, *denoting length* *l*(*ϵ*) *of an interval, there exists a number* *τ* = *τ*(*ϵ*) *in this interval such that* |*x*(*t* + *τ*) − *x*(*t*)| < *ϵ* *for all* t∈R.

Denote the set of almost periodic functions by $AP(R,R^{n})$.

**Definition 2**. *A quaternion-valued function* $x=x^{R}+ix^{I}+jx^{J}+kx^{K}\in C\left(R,Q^{n}\right)$ *is called almost periodic if for every* *ν* ∈ {*R*, *I*, *J*, *K*}: = Λ, $x^{\nu }\in AP\left(R,R^{n}\right)$.

**Definition 3**. [59] *Let* $x\in R^{n}$ *and* *A*(*t*) *be an* *n* × *n* *matrix function on* R. *Then the linear system*

$$\begin{array}{c} x^{\prime }\left(t\right)=A\left(t\right)x\left(t\right),\;\;t\in R \end{array}$$ (2)

*is said to admit an exponential dichotomy on* R *if there exist positive constants* *k*_*i*_, *α*_*i*_, *i* = 1, 2, *projection* *P*, *and the fundamental solution matrix* *X*(*t*) *of* (2), *satisfying*

$$\begin{array}{c} {∥X\left(t\right)PX^{-1}\left(s\right)∥}_{0}\leq k_{1}e^{-\alpha_{1}\left(t-s\right)},\;s,t\in R,\;\;t\geq s,\\ {∥X\left(t\right)\left(I-P\right)X^{-1}\left(s\right)∥}_{0}\leq k_{2}e^{-\alpha_{2}\left(s-t\right)},\;s,t\in R,\;\;t\leq s, \end{array}$$

*where* ‖ ⋅ ‖_0_ *is the matrix norm on* R.

Let us consider the following almost periodic system

$$\begin{array}{c} x^{\prime }\left(t\right)=A\left(t\right)x\left(t\right)+f\left(t\right),\;\;t\in R, \end{array}$$ (3)

where *A*(*t*) is an almost periodic matrix function and *f*(*t*) is an almost periodic vector function.

**Lemma 1**. [59] *If the linear system* (2) *admits an exponential dichotomy, then system* (3) *has a unique almost periodic solution*

$$\begin{array}{c} x\left(t\right)=\int_{-\infty }^{t}X\left(t\right)PX^{-1}\left(s\right)f\left(s\right)\text{d}s-\int_{t}^{+\infty }X\left(t\right)\left(I-P\right)X^{-1}\left(s\right)f\left(s\right)\text{d}s, \end{array}$$

*where* *X*(*t*) *is the fundamental solution matrix of* (2), *I* *denotes the* *n* × *n*-*identity matrix*.

**Lemma 2**. [59] *Let* *a*_*p*_ *be an almost periodic function on* R *and*

$$\begin{array}{c} M\left[a_{p}\right]=\lim_{T\to \infty }\frac{1}{T}\int_{t}^{t+T}a_{p}\left(s\right)\text{d}s>0,\;p=1,2,\ldots n. \end{array}$$

*Then the linear system*

$$\begin{array}{c} x^{\prime }\left(t\right)=\text{diag}\left(-a_{1}\left(t\right),-a_{2}\left(t\right),\ldots ,-a_{n}\left(t\right)\right)x\left(t\right) \end{array}$$

*admits an exponential dichotomy on* R.

Let $x_{pq}=x_{pq}^{R}+ix_{pq}^{I}+jx_{pq}^{J}+kx_{pq}^{K}\in Q$, where $x_{pq}^{R},x_{pq}^{I},x_{pq}^{J},x_{pq}^{K}\in R$. Assume the activity functions f,g,h:Q→Q of (1) can be expressed as

$$\begin{array}{ccc} f(x_{pq}) & = & f^{R}\left(x_{pq}^{R},x_{pq}^{I},x_{pq}^{J},x_{pq}^{K}\right)+if^{I}\left(x_{pq}^{R},x_{pq}^{I},x_{pq}^{J},x_{pq}^{K}\right)\\ & & +jf^{J}\left(x_{pq}^{R},x_{pq}^{I},x_{pq}^{J},x_{pq}^{K}\right)+kf^{K}\left(x_{pq}^{R},x_{pq}^{I},x_{pq}^{J},x_{pq}^{K}\right),\\ g(x_{pq}) & = & g^{R}\left(x_{pq}^{R},x_{pq}^{I},x_{pq}^{J},x_{pq}^{K}\right)+ig^{I}\left(x_{pq}^{R},x_{pq}^{I},x_{pq}^{J},x_{pq}^{K}\right)\\ & & +jg^{J}\left(x_{pq}^{R},x_{pq}^{I},x_{pq}^{J},x_{pq}^{K}\right)+kg^{K}\left(x_{pq}^{R},x_{pq}^{I},x_{pq}^{J},x_{pq}^{K}\right),\\ h(x_{pq}) & = & h^{R}\left(x_{pq}^{R},x_{pq}^{I},x_{pq}^{J},x_{pq}^{K}\right)+ih^{I}\left(x_{pq}^{R},x_{pq}^{I},x_{pq}^{J},x_{pq}^{K}\right)\\ & & +jh^{J}\left(x_{pq}^{R},x_{pq}^{I},x_{pq}^{J},x_{pq}^{K}\right)+kh^{K}\left(x_{pq}^{R},x_{pq}^{I},x_{pq}^{J},x_{pq}^{K}\right), \end{array}$$

where $f^{\nu },g^{\nu },h^{\nu }:R^{4}\to R$, *ν* ∈ Λ, pq∈J and the external input $T_{pq}^{\nu }:R\to Q$ can be expressed as

$$\begin{array}{c} T_{pq}\left(t\right)=T_{pq}^{R}\left(t\right)+iT_{pq}^{I}\left(t\right)+jT_{pq}^{J}\left(t\right)+kT_{pq}^{K}\left(t\right), \end{array}$$

where $T_{pq}^{\nu }:R\to R,\nu \in \Lambda ,pq\in J$.

In the following, for a bounded continuous function, we denote $\bar{f}=\underset{t\in R}{\sup}\left|f\left(t\right)\right|$, $\underline{f}=\underset{t\in R}{\inf}\left|f\left(t\right)\right|$.

In order to overcome the non-commutativity of the quaternion multiplication, according to Hamilton rules, we decompose system (1) into an equivalent real-valued system:

$$\begin{array}{ccc} {\left(x_{pq}^{R}\right)}^{\prime }\left(t\right) & = & -a_{pq}\left(t\right)x_{pq}^{R}\left(t\right)-\sum_{C_{kl}\in N_{r}\left(p,q\right)}B_{pq}^{kl}\left(t\right)(f^{R}\left[t,x\right]x_{pq}^{R}\left(t\right)-f^{I}\left[t,x\right]x_{pq}^{I}\left(t\right)\\ & & -f^{J}\left[t,x\right]x_{pq}^{J}\left(t\right)-f^{K}\left[t,x\right]x_{pq}^{K}\left(t\right))-\sum_{C_{kl}\in N_{s}\left(p,q\right)}C_{pq}^{kl}\left(t\right)(g^{R}\left[t,x\right]x_{pq}^{R}\left(t\right)\\ & & -g^{I}\left[t,x\right]x_{pq}^{I}\left(t\right)-g^{J}\left[t,x\right]x_{pq}^{J}\left(t\right)-g^{K}\left[t,x\right]x_{pq}^{K}\left(t\right))\\ & & -\sum_{C_{kl}\in N_{u}\left(p,q\right)}D_{pq}^{kl}\left(t\right)(\int_{0}^{+\infty }K_{pq}\left(u\right)h^{R}\left[t,u,x\right]\text{d}ux_{pq}^{R}\left(t\right)\\ & & -\int_{0}^{+\infty }K_{pq}\left(u\right)h^{I}\left[t,u,x\right]\text{d}ux_{pq}^{I}\left(t\right)-\int_{0}^{+\infty }K_{pq}\left(u\right)h^{J}\left[t,u,x\right]\text{d}ux_{pq}^{J}\left(t\right)\\ & & -\int_{0}^{+\infty }K_{pq}\left(u\right)h^{K}\left[t,u,x\right]\text{d}ux_{pq}^{K}\left(t\right))+T_{pq}^{R}\left(t\right),\;\;pq\in J, \end{array}$$ (4)

$$\begin{array}{ccc} {\left(x_{pq}^{I}\right)}^{\prime }\left(t\right) & = & -a_{pq}\left(t\right)x_{pq}^{I}\left(t\right)-\sum_{C_{kl}\in N_{r}\left(p,q\right)}B_{pq}^{kl}\left(t\right)(f^{R}\left[t,x\right]x_{pq}^{I}\left(t\right)+f^{I}\left[t,x\right]x_{pq}^{R}\left(t\right)\\ & & +f^{J}\left[t,x\right]x_{pq}^{K}\left(t\right)-f^{K}\left[t,x\right]x_{pq}^{J}\left(t\right))-\sum_{C_{kl}\in N_{s}\left(p,q\right)}C_{pq}^{kl}\left(t\right)(g^{R}\left[t,x\right]x_{pq}^{I}\left(t\right)\\ & & +g^{I}\left[t,x\right]x_{pq}^{R}\left(t\right)+g^{J}\left[t,x\right]x_{pq}^{K}\left(t\right)-g^{K}\left[t,x\right]x_{pq}^{J}\left(t\right))\\ & & -\sum_{C_{kl}\in N_{u}\left(p,q\right)}D_{pq}^{kl}\left(t\right)(\int_{0}^{+\infty }K_{pq}\left(u\right)h^{R}\left[t,u,x\right]\text{d}ux_{pq}^{I}\left(t\right)\\ & & +\int_{0}^{+\infty }K_{pq}\left(u\right)h^{I}\left[t,u,x\right]\text{d}ux_{pq}^{R}\left(t\right)+\int_{0}^{+\infty }K_{pq}\left(u\right)h^{J}\left[t,u,x\right]\text{d}ux_{pq}^{K}\left(t\right)\\ & & -\int_{0}^{+\infty }K_{pq}\left(u\right)h^{K}\left[t,u,x\right]\text{d}ux_{pq}^{J}\left(t\right))+T_{pq}^{I}\left(t\right),\;\;pq\in J, \end{array}$$ (5)

$$\begin{array}{ccc} {\left(x_{pq}^{J}\right)}^{\prime }\left(t\right) & = & -a_{pq}\left(t\right)x_{pq}^{J}\left(t\right)-\sum_{C_{kl}\in N_{r}\left(p,q\right)}B_{pq}^{kl}\left(t\right)(f^{R}\left[t,x\right]x_{pq}^{J}\left(t\right)-f^{I}\left[t,x\right]x_{pq}^{K}\left(t\right)\\ & & +f^{J}\left[t,x\right]x_{pq}^{R}\left(t\right)+f^{K}\left[t,x\right]x_{pq}^{I}\left(t\right))-\sum_{C_{kl}\in N_{s}\left(p,q\right)}C_{pq}^{kl}\left(t\right)(g^{R}\left[t,x\right]x_{pq}^{J}\left(t\right)\\ & & -g^{I}\left[t,x\right]x_{pq}^{K}\left(t\right)+g^{J}\left[t,x\right]x_{pq}^{R}\left(t\right)+g^{K}\left[t,x\right]x_{pq}^{I}\left(t\right))\\ & & -\sum_{C_{kl}\in N_{u}\left(p,q\right)}D_{pq}^{kl}\left(t\right)(\int_{0}^{+\infty }K_{pq}\left(u\right)h^{R}\left[t,u,x\right]\text{d}ux_{pq}^{J}\left(t\right)\\ & & -\int_{0}^{+\infty }K_{pq}\left(u\right)h^{I}\left[t,u,x\right]\text{d}ux_{pq}^{K}\left(u\right)+\int_{0}^{+\infty }K_{pq}\left(u\right)h^{J}\left[t,u,x\right]\text{d}ux_{pq}^{R}\left(t\right)\\ & & +\int_{0}^{+\infty }K_{pq}\left(u\right)h^{K}\left[t,u,x\right]\text{d}ux_{pq}^{I}\left(t\right))+T_{pq}^{J}\left(t\right),\;\;pq\in J, \end{array}$$ (6)

$$\begin{array}{ccc} {\left(x_{pq}^{K}\right)}^{\prime }\left(t\right) & = & -a_{pq}\left(t\right)x_{pq}^{K}\left(t\right)-\sum_{C_{kl}\in N_{r}\left(p,q\right)}B_{pq}^{kl}\left(t\right)(f^{R}\left[t,x\right]x_{pq}^{K}\left(t\right)+f^{I}\left[t,x\right]x_{pq}^{J}\left(t\right)\\ & & -f^{J}\left[t,x\right]x_{pq}^{I}\left(t\right)+f^{K}\left[t,x\right]x_{pq}^{R}\left(t\right))-\sum_{C_{kl}\in N_{s}\left(p,q\right)}C_{pq}^{kl}\left(t\right)(g^{R}\left[t,x\right]x_{pq}^{K}\left(t\right)\\ & & +g^{I}\left[t,x\right]x_{pq}^{J}\left(t\right)-g^{J}\left[t,x\right]x_{pq}^{I}\left(t\right)+g^{K}\left[t,x\right]x_{pq}^{R}\left(t\right))\\ & & -\sum_{C_{kl}\in N_{u}\left(p,q\right)}D_{pq}^{kl}\left(t\right)(\int_{0}^{+\infty }K_{pq}\left(u\right)h^{R}\left[t,u,x\right]\text{d}ux_{pq}^{K}\left(t\right)\\ & & +\int_{0}^{+\infty }K_{pq}\left(u\right)h^{I}\left[t,u,x\right]\text{d}ux_{pq}^{J}\left(t\right)-\int_{0}^{+\infty }K_{pq}\left(u\right)h^{J}\left[t,u,x\right]\text{d}ux_{pq}^{I}\left(t\right)\\ & & +\int_{0}^{+\infty }K_{pq}\left(u\right)h^{K}\left[t,u,x\right]\text{d}ux_{pq}^{R}\left(t\right))+T_{pq}^{K}\left(t\right),\;\;pq\in J, \end{array}$$ (7)

where $f^{\nu }\left[t,x\right]≜f^{\nu }\left(x_{kl}^{R}\left(t\right),x_{kl}^{I}\left(t\right),x_{kl}^{J}\left(t\right),x_{kl}^{K}\left(t\right)\right),$ $g^{\nu }\left[t,x\right]≜g^{\nu }\left(x_{kl}^{R}\left(t-\tau \left(t\right)\right),x_{kl}^{I}\left(t-\tau \left(t\right)\right),x_{kl}^{J}\left(t-\tau \left(t\right)\right),x_{kl}^{K}\left(t-\tau \left(t\right)\right)\right),$ $h^{\nu }\left[t,u,x\right]≜h^{\nu }\left(x_{kl}^{R}\left(t-u\right),x_{kl}^{I}\left(t-u\right),x_{kl}^{J}\left(t-u\right),x_{kl}^{K}\left(t-u\right)\right).$

Denote

$$\begin{array}{c} F\left[t,x\right]=\left(\begin{array}{cccc} f^{R}\left[t,x\right] & -f^{I}\left[t,x\right] & -f^{J}\left[t,x\right] & -f^{K}\left[t,x\right]\\ f^{I}\left[t,x\right] & f^{R}\left[t,x\right] & -f^{K}\left[t,x\right] & f^{J}\left[t,x\right]\\ f^{J}\left[t,x\right] & f^{K}\left[t,x\right] & f^{R}\left[t,x\right] & -f^{I}\left[t,x\right]\\ f^{K}\left[t,x\right] & -f^{J}\left[t,x\right] & f^{I}\left[t,x\right] & f^{R}\left[t,x\right] \end{array}\right), \end{array}$$

$$\begin{array}{c} G\left[t,x\right]=\left(\begin{array}{cccc} g^{R}\left[t,x\right] & -g^{I}\left[t,x\right] & -g^{J}\left[t,x\right] & -g^{K}\left[t,x\right]\\ g^{I}\left[t,x\right] & g^{R}\left[t,x\right] & -g^{K}\left[t,x\right] & g^{J}\left[t,x\right]\\ g^{J}\left[t,x\right] & g^{K}\left[t,x\right] & g^{R}\left[t,x\right] & -g^{I}\left[t,x\right]\\ g^{K}\left[t,x\right] & -g^{J}\left[t,x\right] & g^{I}\left[t,x\right] & g^{R}\left[t,x\right] \end{array}\right), \end{array}$$

$$\begin{array}{c} H\left[t,u,x\right]=\left(\begin{array}{cccc} h^{R}\left[t,u,x\right] & -h^{I}\left[t,u,x\right] & -h^{J}\left[t,u,x\right] & -h^{K}\left[t,u,x\right]\\ h^{I}\left[t,u,x\right] & h^{R}\left[t,u,x\right] & -h^{K}\left[t,u,x\right] & h^{J}\left[t,u,x\right]\\ h^{J}\left[t,u,x\right] & h^{K}\left[t,u,x\right] & h^{R}\left[t,u,x\right] & -h^{I}\left[t,u,x\right]\\ h^{K}\left[t,u,x\right] & -h^{J}\left[t,u,x\right] & h^{I}\left[t,u,x\right] & h^{R}\left[t,u,x\right] \end{array}\right), \end{array}$$

$$\begin{array}{c} X_{pq}=\left(\begin{array}{c} x_{pq}^{R}\\ x_{pq}^{I}\\ x_{pq}^{J}\\ x_{pq}^{K} \end{array}\right),\;\;T_{pq}=\left(\begin{array}{c} T_{pq}^{R}\\ T_{pq}^{I}\\ T_{pq}^{J}\\ T_{pq}^{K} \end{array}\right),\;\; \end{array}$$

Applying (4)–(7), we obtain an equivalent real-valued system of the quaternion-valued system (1) as follows:

$$\begin{array}{ccc} X_{pq}^{\prime }\left(t\right) & = & -a_{pq}\left(t\right)X_{pq}\left(t\right)-\sum_{C_{kl}\in N_{r}\left(p,q\right)}B_{pq}^{kl}\left(t\right)F\left[t,x\right]X_{pq}\left(t\right)\\ & & -\sum_{C_{kl}\in N_{s}\left(p,q\right)}C_{pq}^{kl}\left(t\right)G\left[t,x\right]X_{pq}\left(t\right)+T_{pq}\left(t\right)\\ & & -\sum_{C_{kl}\in N_{u}\left(p,q\right)}D_{pq}^{kl}\left(t\right)\int_{0}^{+\infty }K_{pq}\left(u\right)H\left[t,u,x\right]\text{d}uX_{pq}\left(t\right),\;\;pq\in J\;\;\;\; \end{array}$$ (8)

with the initial conditions:

$$\begin{array}{c} X_{pq}\left(s\right)=\phi_{pq}\left(s\right),s\in \left(-\infty ,0\right],\;\;pq\in J, \end{array}$$

where $\phi_{pq}={\left(\varphi_{pq}^{R},\varphi_{pq}^{I},\varphi_{pq}^{J},\varphi_{pq}^{K}\right)}^{T}$, $\varphi_{pq}^{\nu }\in C\left(\left(-\infty ,0\right],R\right),\nu \in \Lambda .$

In what follows, we regard (1) as the drive system, and the corresponding response system is expressed as

$$\begin{array}{ccc} y_{pq}^{\prime }\left(t\right) & = & -a_{pq}\left(t\right)y_{pq}\left(t\right)-\sum_{C_{kl}\in N_{r}\left(p,q\right)}B_{pq}^{kl}\left(t\right)f\left(y_{kl}\left(t\right)\right)y_{pq}\left(t\right)\\ & & -\sum_{C_{kl}\in N_{s}\left(p,q\right)}C_{pq}^{kl}\left(t\right)g\left(y_{kl}\left(t-\tau \left(t\right)\right)\right)y_{pq}\left(t\right)\\ & & -\sum_{C_{kl}\in N_{u}\left(p,q\right)}D_{pq}^{kl}\left(t\right)\int_{0}^{+\infty }K_{pq}\left(u\right)h\left(y_{kl}\left(t-u\right)\right)\text{d}uy_{pq}\left(t\right)\\ & & +T_{pq}\left(t\right)+U_{pq}\left(t\right),\;pq\in J, \end{array}$$ (9)

where $y_{pq}\left(t\right)=y_{pq}^{R}\left(t\right)+iy_{pq}^{I}\left(t\right)+jy_{pq}^{J}\left(t\right)+ky_{pq}^{K}\left(t\right)$ denotes the state of the response system, $U_{pq}\left(t\right)=U_{pq}^{R}\left(t\right)+iU_{pq}^{I}\left(t\right)+jU_{pq}^{J}\left(t\right)+kU_{pq}^{K}\left(t\right)$ is a state-feedback controller, the rest notations are the same as those in system (1) and the initial condition is

$$\begin{array}{c} y_{pq}\left(s\right)=\psi_{pq}\left(s\right),\;\;s\in \left(-\infty ,0\right],\;\;pq\in J, \end{array}$$

where $\psi_{pq}\left(s\right)=\psi_{pq}^{R}\left(s\right)+i\psi_{pq}^{I}\left(s\right)+\psi_{pq}^{J}\left(s\right)+\psi_{pq}^{K}\left(s\right)$ are quaternion-valued bounded continuous functions on (−∞, 0].

Denote *z*_*pq*_(*t*) = *y*_*pq*_(*t*) − *x*_*pq*_(*t*), subtracting (1) from (9) yields the following error system:

$$\begin{array}{ccc} z_{pq}^{\prime }\left(t\right) & = & -a_{pq}\left(t\right)z_{pq}\left(t\right)-\sum_{C_{kl}\in N_{r}\left(p,q\right)}B_{pq}^{kl}\left(t\right)F\left(z_{kl}\left(t\right)\right)z_{pq}\left(t\right)\\ & & -\sum_{C_{kl}\in N_{s}\left(p,q\right)}C_{pq}^{kl}\left(t\right)G\left(z_{kl}\left(t-\tau \left(t\right)\right)\right)z_{pq}\left(t\right)\\ & & -\sum_{C_{kl}\in N_{u}\left(p,q\right)}D_{pq}^{kl}\left(t\right)\int_{0}^{+\infty }K_{pq}\left(u\right)H\left(z_{kl}\left(t-u\right)\right)\text{d}uz_{pq}\left(t\right)\\ & & +U_{pq}\left(t\right),\;pq\in J, \end{array}$$ (10)

where *F*(*z*_*kl*_(*t*))*z*_*pq*_(*t*) = *f*(*y*_*kl*_(*t*))*y*_*pq*_(*t*) − *f*(*x*_*kl*_(*t*))*x*_*pq*_(*t*), *G*(*z*_*kl*_(*t* − *τ*(*t*)))*z*_*pq*_(*t*) = *g*(*y*_*kl*_(*t* − *τ*(*t*)))*y*_*pq*_(*t*) − *g*(*x*_*kl*_(*t* − *τ*(*t*)))*x*_*pq*_(*t*), *H*(*z*_*kl*_(*t* − *u*))*z*_*pq*_(*t*) = *h*(*y*_*kl*_(*t* − *u*))*y*_*pq*_(*t*) − *h*(*x*_*kl*_(*t* − *u*))*x*_*pq*_(*t*).

In order to show the almost periodic synchronization of the drive-response system, we design the state-feedback controller as follows:

$$\begin{array}{c} U_{pq}\left(t\right)=-d_{pq}\left(t\right)z_{pq}\left(t\right)-\sum_{C_{kl}\in N_{v}\left(p,q\right)}E_{pq}^{kl}\left(t\right)W\left(z_{kl}\left(t-\delta \left(t\right)\right)\right)z_{pq}\left(t\right),\;\;pq\in J, \end{array}$$

where *W*(*z*_*kl*_(*t* − *δ*(*t*))) = *w*(*y*_*kl*_(*t* − *δ*(*t*)))*y*_*pq*_(*t*) − *w*(*x*_*kl*_(*t* − *δ*(*t*)))*x*_*pq*_(*t*).

**Definition 4**. *The response system* (9) *and the drive system* (1) *are said to be globally exponentially synchronized, if there exist positive constants* *M* > 0 *and* λ > 0 *such that*

$$\begin{array}{c} {∥y(t)-x(t)∥}_{0}\leq M∥\psi -\varphi ∥e^{-\lambda t}, \end{array}$$

*where*

$$\begin{array}{c} {∥y(t)-x(t)∥}_{0}=\max_{pq\in J,\nu \in \Lambda }\left\{\right|y_{pq}^{\nu }\left(t\right)-x_{pq}^{\nu }\left(t\right)\left|\right\}, \end{array}$$

$$\begin{array}{c} ∥\psi -\varphi ∥=\max_{pq\in J,\nu \in \Lambda }\{\underset{t\in R}{\sup}|\psi_{pq}^{\nu }\left(t\right)-\varphi_{pq}^{\nu }\left(t\right)\left|\right\}. \end{array}$$

Analogously, one can decompose (10) into the following real-valued system:

$$\begin{array}{ccc} {\left(z_{pq}^{R}\right)}^{\prime }\left(t\right) & = & -a_{pq}\left(t\right)z_{pq}^{R}\left(t\right)-\sum_{C_{kl}\in N_{r}\left(p,q\right)}B_{pq}^{kl}\left(t\right)\{(f^{R}\left[t,y\right]y_{pq}^{R}\left(t\right)-f^{R}\left[t,x\right]x_{pq}^{R}\left(t\right))\\ & & -\left(f^{I}\left[t,y\right]y_{pq}^{I}\left(t\right)-f^{I}\left[t,x\right]x_{pq}^{I}\left(t\right)\right)-\left(f^{J}\left[t,y\right]y_{pq}^{J}\left(t\right)-f^{J}\left[t,x\right]x_{pq}^{J}\left(t\right)\right)\\ & & -\left(f^{K}\left[t,y\right]y_{pq}^{K}\left(t\right)-f^{K}\left[t,x\right]x_{pq}^{K}\left(t\right))\right\}+\sum_{C_{kl}\in N_{s}\left(p,q\right)}C_{pq}^{kl}\left(t\right)\{(g^{R}\left[t,y\right]\\ & & \times y_{pq}^{R}\left(t\right)-g^{R}\left[t,x\right]x_{pq}^{R}\left(t\right))-\left(g^{I}\left[t,y\right]y_{pq}^{I}\left(t\right)-g^{I}\left[t,x\right]x_{pq}^{I}\left(t\right)\right)\\ & & -\left(g^{J}\left[t,y\right]y_{pq}^{J}\left(t\right)-g^{J}\left[t,x\right]x_{pq}^{J}\left(t\right)\right)-\left(g^{K}\left[t,y\right]y_{pq}^{K}\left(t\right)-g^{K}\left[t,x\right]x_{pq}^{K}\left(t\right))\right\}\\ & & -\sum_{C_{kl}\in N_{u}\left(p,q\right)}D_{pq}^{kl}\left(t\right)\{\int_{0}^{+\infty }K_{pq}\left(u\right)(h^{R}\left[t,u,y\right]y_{pq}^{R}\left(t\right)\\ & & -h^{R}\left[t,u,x\right]x_{pq}^{R}\left(t\right))\text{d}u-\int_{0}^{+\infty }K_{pq}\left(u\right)(h^{I}\left[t,u,y\right]y_{pq}^{I}\left(t\right)\\ & & -h^{I}\left[t,u,x\right]x_{pq}^{I}\left(t\right))\text{d}u-\int_{0}^{+\infty }K_{pq}\left(u\right)(h^{J}\left[t,u,y\right]y_{pq}^{J}\left(t\right)\\ & & -h^{J}\left[t,u,x\right]x_{pq}^{J}\left(t\right))\text{d}u-\int_{0}^{+\infty }K_{pq}\left(u\right)(h^{K}\left[t,u,y\right]y_{pq}^{K}\left(t\right)\\ & & -h^{K}\left[t,u,x\right]x_{pq}^{K}\left(t\right))\text{d}u\}-d_{pq}\left(t\right)z_{pq}^{R}\left(t\right)-\sum_{C_{kl}\in N_{v}\left(p,q\right)}E_{pq}^{kl}\left(t\right)\\ & & \times \{W^{R}\left[t,z\right]z_{pq}^{R}\left(t\right)-W^{I}\left[t,z\right]z_{pq}^{I}\left(t\right)-W^{J}\left[t,z\right]z_{pq}^{J}\left(t\right)\\ & & -W^{K}\left[t,z\right]z_{pq}^{K}\left(t\right)\}, \end{array}$$ (11)

$$\begin{array}{ccc} {\left(z_{pq}^{I}\right)}^{\prime }\left(t\right) & = & -a_{pq}\left(t\right)z_{pq}^{I}\left(t\right)-\sum_{C_{kl}\in N_{r}\left(p,q\right)}B_{pq}^{kl}\left(t\right)\{(f^{R}\left[t,y\right]y_{pq}^{I}\left(t\right)-\left(f^{R}\left[t,x\right]x_{pq}^{I}\left(t\right)\right)\\ & & +\left(f^{I}\left[t,y\right]y_{pq}^{R}\left(t\right)-f^{I}\left[t,x\right]x_{pq}^{R}\left(t\right)\right)+\left(f^{J}\left[t,y\right]y_{pq}^{K}\left(t\right)-f^{J}\left[t,x\right]x_{pq}^{K}\left(t\right)\right)\\ & & -\left(f^{K}\left[t,y\right]y_{pq}^{J}\left(t\right)-f^{K}\left[t,x\right]x_{pq}^{J}\left(t\right))\right\}-\sum_{C_{kl}\in N_{s}\left(p,q\right)}C_{pq}^{kl}\left(t\right)\{(g^{R}\left[t,y\right]\\ & & \times y_{pq}^{I}\left(t\right)-g^{R}\left[t,x\right]x_{pq}^{I}\left(t\right))+\left(g^{I}\left[t,y\right]y_{pq}^{R}\left(t\right)-g^{I}\left[t,x\right]x_{pq}^{R}\left(t\right)\right)\\ & & +\left(g^{J}\left[t,y\right]y_{pq}^{K}\left(t\right)-g^{J}\left[t,x\right]x_{pq}^{K}\left(t\right)\right)-\left(g^{K}\left[t,y\right]y_{pq}^{J}\left(t\right)-g^{K}\left[t,x\right]x_{pq}^{J}\left(t\right))\right\}\\ & & -\sum_{C_{kl}\in N_{u}\left(p,q\right)}D_{pq}^{kl}\left(t\right)\{\int_{0}^{+\infty }K_{pq}\left(u\right)(h^{R}\left[t,u,y\right]y_{pq}^{I}\left(t\right)\\ & & -h^{R}\left[t,u,x\right]x_{pq}^{I}\left(t\right))\text{d}u+\int_{0}^{+\infty }K_{pq}\left(u\right)(h^{I}\left[t,u,y\right]y_{pq}^{R}\left(t\right)\\ & & -h^{I}\left[t,u,x\right]x_{pq}^{R}\left(t\right))\text{d}u+\int_{0}^{+\infty }K_{pq}\left(u\right)(h^{J}\left[t,u,y\right]y_{pq}^{K}\left(t\right)\\ & & -h^{J}\left[t,u,x\right]x_{pq}^{K}\left(t\right))\text{d}u-\int_{0}^{+\infty }K_{pq}\left(u\right)(h^{K}\left[t,u,y\right]y_{pq}^{J}\left(t\right)\\ & & -h^{K}\left[t,u,x\right]x_{pq}^{J}\left(t\right))\text{d}u\}-d_{pq}\left(t\right)z_{pq}^{I}\left(t\right)-\sum_{C_{kl}\in N_{v}\left(p,q\right)}E_{pq}^{kl}\left(t\right)\\ & & \times \{W^{R}\left[t,z\right]z_{pq}^{I}\left(t\right)+W^{I}\left[t,z\right]z_{pq}^{R}\left(t\right)+W^{J}\left[t,z\right]z_{pq}^{K}\left(t\right)\\ & & -W^{K}\left[t,z\right]z_{pq}^{J}\left(t)\right\}, \end{array}$$ (12)

$$\begin{array}{ccc} {\left(z_{pq}^{J}\right)}^{\prime }\left(t\right) & = & -a_{pq}\left(t\right)z_{pq}^{J}\left(t\right)-\sum_{C_{kl}\in N_{r}\left(p,q\right)}B_{pq}^{kl}\left(t\right)\{(f^{R}\left[t,y\right]y_{pq}^{J}\left(t\right)-f^{R}\left[t,x\right]x_{pq}^{J}\left(t\right))\\ & & -\left(f^{I}\left[t,y\right]y_{pq}^{K}\left(t\right)-f^{I}\left[t,x\right]x_{pq}^{K}\left(t\right)\right)+\left(f^{J}\left[t,y\right]y_{pq}^{R}\left(t\right)-f^{J}\left[t,x\right]x_{pq}^{R}\left(t\right)\right)\\ & & +\left(f^{K}\left[t,y\right]y_{pq}^{I}\left(t\right)-f^{K}\left[t,x\right]x_{pq}^{I}\left(t\right))\right\}-\sum_{C_{kl}\in N_{s}\left(p,q\right)}C_{pq}^{kl}\left(t\right)\{(g^{R}\left[t,y\right]\\ & & \times y_{pq}^{J}\left(t\right)-g^{R}\left[t,x\right]x_{pq}^{J}\left(t\right))-\left(g^{I}\left[t,y\right]y_{pq}^{K}\left(t\right)-g^{I}\left[t,x\right]x_{pq}^{K}\left(t\right)\right)\\ & & +\left(g^{J}\left[t,y\right]y_{pq}^{R}\left(t\right)-g^{J}\left[t,x\right]x_{pq}^{R}\left(t\right)\right)+\left(g^{K}\left[t,y\right]y_{pq}^{I}\left(t\right)-g^{K}\left[t,x\right]x_{pq}^{I}\left(t\right))\right\}\\ & & -\sum_{C_{kl}\in N_{u}\left(p,q\right)}D_{pq}^{kl}\left(t\right)\{\int_{0}^{+\infty }K_{pq}\left(u\right)(h^{R}\left[t,u,y\right]y_{pq}^{J}\left(t\right)\\ & & -h^{R}\left[t,u,x\right]x_{pq}^{J}\left(t\right))\text{d}u-\int_{0}^{+\infty }K_{pq}\left(u\right)(h^{I}\left[t,u,y\right]y_{pq}^{K}\left(t\right)\\ & & -h^{I}\left[t,u,x\right]x_{pq}^{K}\left(t\right))\text{d}u+\int_{0}^{+\infty }K_{pq}\left(u\right)(h^{J}\left[t,u,y\right]y_{pq}^{R}\left(t\right)\\ & & -h^{J}\left[t,u,x\right]x_{pq}^{R}\left(t\right))\text{d}u+\int_{0}^{+\infty }K_{pq}\left(u\right)(h^{K}\left[t,u,y\right]\\ & & \times y_{pq}^{I}\left(t\right)-h^{K}\left[t,u,x\right]z_{pq}^{I}\left(t\right))\text{d}u\}-d_{pq}\left(t\right)z_{pq}^{J}\left(t\right)-\sum_{C_{kl}\in N_{v}\left(p,q\right)}E_{pq}^{kl}\left(t\right)\\ & & \times \{W^{R}\left[t,z\right]z_{pq}^{J}\left(t\right)-W^{I}\left[t,z\right]z_{pq}^{K}\left(t\right)+W^{J}\left[t,z\right]z_{pq}^{R}\left(t\right)\\ & & +W^{K}\left[t,z\right]z_{pq}^{I}\left(t\right)\}, \end{array}$$ (13)

$$\begin{array}{ccc} {\left(z_{pq}^{K}\right)}^{\prime }\left(t\right) & = & -a_{pq}\left(t\right)z_{pq}^{K}\left(t\right)-\sum_{C_{kl}\in N_{r}\left(p,q\right)}B_{pq}^{kl}\left(t\right)\{(f^{R}\left[t,y\right]y_{pq}^{K}\left(t\right)-f^{R}\left[t,x\right]x_{pq}^{K}\left(t\right))\\ & & +\left(f^{I}\left[t,y\right]y_{pq}^{J}\left(t\right)-f^{I}\left[t,x\right]x_{pq}^{J}\left(t\right)\right)-\left(f^{J}\left[t,y\right]y_{pq}^{I}\left(t\right)-f^{J}\left[t,x\right]x_{pq}^{I}\left(t\right)\right)\\ & & +\left(f^{K}\left[t,y\right]y_{pq}^{R}\left(t\right)-f^{K}\left[t,x\right]x_{pq}^{R}\left(t\right))\right\}-\sum_{C_{kl}\in N_{s}\left(p,q\right)}C_{pq}^{kl}\left(t\right)\{(g^{R}\left[t,y\right]\\ & & \times y_{pq}^{K}\left(t\right)-g^{R}\left[t,x\right]x_{pq}^{K}\left(t\right))+\left(g^{I}\left[t,y\right]y_{pq}^{J}\left(t\right)-g^{I}\left[t,x\right]x_{pq}^{J}\left(t\right)\right)\\ & & -\left(g^{J}\left[t,y\right]y_{pq}^{I}\left(t\right)-g^{J}\left[t,x\right]x_{pq}^{I}\left(t\right)\right)+\left(g^{K}\left[t,y\right]y_{pq}^{R}\left(t\right)-g^{K}\left[t,x\right]x_{pq}^{R}\left(t\right))\right\}\\ & & -\sum_{C_{kl}\in N_{u}\left(p,q\right)}D_{pq}^{kl}\left(t\right)\{\int_{0}^{+\infty }K_{pq}\left(u\right)(h^{R}\left[t,u,y\right]y_{pq}^{K}\left(t\right)\\ & & -h^{R}\left[t,u,x\right]x_{pq}^{K}\left(t\right))\text{d}u+\int_{0}^{+\infty }K_{pq}\left(u\right)(h^{I}\left[t,u,y\right]y_{pq}^{J}\left(t\right)\\ & & -h^{I}\left[t,u,x\right]x_{pq}^{J}\left(t\right))\text{d}u-\int_{0}^{+\infty }K_{pq}\left(u\right)(h^{J}\left[t,u,y\right]y_{pq}^{I}\left(t\right)\\ & & -h^{J}\left[t,u,x\right]x_{pq}^{I}\left(t\right))\text{d}u+\int_{0}^{+\infty }K_{pq}\left(u\right)(h^{K}\left[t,u,y\right]\\ & & \times y_{pq}^{R}\left(t\right)-h^{K}\left[t,u,x\right]x_{pq}^{R}\left(t\right))\text{d}u\}-d_{pq}\left(t\right)z_{pq}^{K}\left(t\right)-\sum_{C_{kl}\in N_{v}\left(p,q\right)}E_{pq}^{kl}\left(t\right)\\ & & \times \{W^{R}\left[t,z\right]z_{pq}^{K}\left(t\right)+W^{I}\left[t,z\right]z_{pq}^{J}\left(t\right)-W^{J}\left[t,z\right]z_{pq}^{I}\left(t\right)\\ & & +W^{K}\left[t,z\right]z_{pq}^{R}\left(t\right)\}, \end{array}$$ (14)

where $pq\in J,W^{\nu }\left[t,z\right]≜W^{\nu }\left(z_{kl}^{R}\left(t-\delta \left(t\right)\right),z_{kl}^{I}\left(t-\delta \left(t\right)\right),z_{kl}^{J}\left(t-\delta \left(t\right)\right),z_{kl}^{K}\left(t-\delta \left(t\right)\right)\right),\nu \in \Lambda .$

**Remark 1.** *If* $X_{pq}\left(t\right)=\left(x_{pq}^{R}\left(t\right),x_{pq}^{I}\left(t\right),x_{pq}^{J}\left(t\right),x_{pq}^{K}\left(t\right)\right)$ *is a solution to system* (8), *then* $x_{pq}\left(t\right)=x_{pq}^{R}\left(t\right)+ix_{pq}^{I}\left(t\right)+jx_{pq}^{J}\left(t\right)+kx_{pq}^{K}\left(t\right)$ (*pq* ∈ *J*) *must be a solution to system* (1). *Thus, the problem of finding an almost periodic solution for* (1) *is reduced to finding it for system* (8). *For studying the synchronization of* (1) *and* (9), *we just need to consider the exponential stability of system* (11)–(14).

Throughout the paper, we assume the following conditions:

(*A*_1_) For pq,kl∈J, *ν* ∈ Λ, $a_{pq}\in AP\left(R,R^{+}\right)$ with *M*[*a*_*pq*_] > 0, $T_{pq}^{\nu }\in AP\left(R,R\right)$, $d_{pq},B_{pq}^{kl},C_{pq}^{kl},D_{pq}^{kl},E_{pq}^{kl},\tau ,\delta \in AP\left(R,R^{+}\right)$, and 1 − *α* > 0, 1 − *β* > 0, where $\alpha =\underset{t\in R}{\sup}\tau^{\prime }\left(t\right),\beta =\underset{t\in R}{\sup}\delta^{\prime }\left(t\right)$.
(*A*_2_) For *ν* ∈ Λ, *f*^*ν*^, *g*^*ν*^, *h*^*ν*^, $p^{\nu }\in C\left(R,R\right)$ and for any $u^{\nu },v^{\nu }\in R$, there exist positive constants $L_{f}^{\nu }$, $L_{g}^{\nu }$, $L_{h}^{\nu }$, $L_{w}^{\nu }$, $M_{f}^{\nu },M_{g}^{\nu },M_{h}^{\nu },M_{w}^{\nu }$ such that
  $$\begin{array}{ccc} |f^{\nu }\left(u^{R},u^{I},u^{J},u^{K}\right)-f^{\nu }\left(v^{R},v^{I},v^{J},v^{K}\right)| & \leq & L_{f}^{R}|u^{R}-v^{R}|+L_{f}^{I}\left|u^{I}-v^{I}\right|\\ & & +L_{f}^{J}|u^{J}-v^{J}|+L_{f}^{K}\left|u^{K}-v^{K}\right|,\\ |g^{\nu }\left(u^{R},u^{I},u^{J},u^{K}\right)-g^{\nu }\left(v^{R},v^{I},v^{J},v^{K}\right)| & \leq & L_{g}^{R}|u^{R}-v^{R}|+L_{g}^{I}\left|u^{I}-v^{I}\right|\\ & & +L_{g}^{J}|u^{J}-v^{J}|+L_{g}^{K}\left|u^{K}-v^{K}\right|,\\ |h^{\nu }\left(u^{R},u^{I},u^{J},u^{K}\right)-h^{\nu }\left(v^{R},v^{I},v^{J},v^{K}\right)| & \leq & L_{h}^{R}|u^{R}-v^{R}|+L_{h}^{I}\left|u^{I}-v^{I}\right|\\ & & +L_{h}^{J}|u^{J}-v^{J}|+L_{h}^{K}\left|u^{K}-v^{K}\right|,\\ |w^{\nu }\left(u^{R},u^{I},u^{J},u^{K}\right)-w^{\nu }\left(v^{R},v^{I},v^{J},v^{K}\right)| & \leq & L_{w}^{R}|u^{R}-v^{R}|+L_{w}^{I}\left|u^{I}-v^{I}\right|\\ & & +L_{w}^{J}|u^{J}-v^{J}|+L_{w}^{K}\left|u^{K}-v^{K}\right|, \end{array}$$
  and
  $$\begin{array}{ccc} & & f^{\nu }\left(\left(u^{R},u^{I},u^{J},u^{K}\right)\right)\leq M_{f}^{\nu },\;g^{\nu }\left(u^{R},u^{I},u^{J},u^{K}\right)\leq M_{g}^{\nu },\\ & & h^{\nu }\left(u^{R},u^{I},u^{J},u^{K}\right)\leq M_{h}^{\nu },\;w^{\nu }\left(u^{R},u^{I},u^{J},u^{K}\right)\leq M_{w}^{\nu }. \end{array}$$
(*A*_3_) For pq∈J, the delay kernels $K_{pq}:\left[0,\infty \right)\to R$ are continuous and |*K*_*pq*_(*t*)|*e*^λ*t*^ are integrable on [0,∞) for certain positive constant λ.

## Main results

In this section, we establish the sufficient conditions for the existence of almost periodic solutions of system (1), and the sufficient conditions for the global exponential synchronization of the drive system (1) and the response system (9).

Denote $\left\{x_{pq}\right\}=\left\{\left(x_{pq}^{R},x_{pq}^{I},x_{pq}^{J},x_{pq}^{K}\right)\right\}$, where $\left(x_{pq}^{R},x_{pq}^{I},x_{pq}^{J},x_{pq}^{K}\right)=\left(x_{11}^{R},\ldots ,x_{1n}^{R},\ldots ,x_{p1}^{R},\ldots ,x_{pn}^{R},\ldots ,x_{mn}^{R},\ldots ,x_{11}^{R},\ldots ,x_{1n}^{K},\ldots ,x_{p1}^{K},\ldots ,x_{pn}^{K},\ldots ,x_{mn}^{K}\right)$. For $x=\left\{x_{pq}\right\}\in R^{4mn}$, we define its norm as $∥x∥=\max_{pq\in J}\{\max_{\nu \in \Lambda }|x_{pq}^{\nu }|\}$.

Set $Y=\{\varphi =\left\{\varphi_{pq}\right\}|\varphi \in AP\left(R,R^{4mn}\right)\}$. Y is a Banach space when equipped with the norm $∥\varphi ∥=\max_{pq\in J}\{\max_{\nu \in J}\{\underset{t\in R}{\sup}|\varphi_{pq}^{\nu }\left(t\right)|\}\}$.

**Theorem 1.** *Under assumptions* (*A*_1_)-(*A*_3_), *and*

(*A*_4_) *there exists a positive constant* *κ* *such that*
  $$\begin{array}{c} \vartheta =\max_{pq\in J}\left\{\max_{\nu \in \Lambda }\left\{\frac{\vartheta_{pq}\kappa +{\bar{T}}_{pq}^{\nu }}{{\underline{a}}_{pq}}\right\}\right\}\leq \kappa ,\;\mu =\max_{pq\in J}\left\{\frac{\mu_{pq}}{{\underline{a}}_{pq}}\right\}<1, \end{array}$$
  *where*
  $$\begin{array}{ccc} \vartheta_{pq} & = & \sum_{C_{kl}\in N_{r}\left(p,q\right)}{\bar{B}}_{pq}^{kl}\left(M_{f}^{R}+M_{f}^{I}+M_{f}^{J}+M_{f}^{K}\right)+\sum_{C_{kl}\in N_{s}\left(p,q\right)}{\bar{C}}_{pq}^{kl}(M_{g}^{R}\\ & & +M_{g}^{I}+M_{g}^{J}+M_{g}^{K})+\sum_{C_{kl}\in N_{u}\left(p,q\right)}{\bar{D}}_{pq}^{kl}\int_{0}^{+\infty }|K_{pq}\left(u\right)|\text{d}u(M_{h}^{R}\\ & & +M_{h}^{I}+M_{h}^{J}+M_{h}^{K}), \end{array}$$
  $$\begin{array}{ccc} \mu_{pq} & = & \sum_{C_{kl}\in N_{r}\left(p,q\right)}{\bar{B}}_{pq}^{kl}[M_{f}^{R}+M_{f}^{I}+M_{f}^{J}+M_{f}^{K}+4\kappa (L_{f}^{R}+L_{f}^{I}\\ & & +L_{f}^{J}+L_{f}^{K})]+\sum_{C_{kl}\in N_{s}\left(p,q\right)}{\bar{C}}_{pq}^{kl}[M_{g}^{R}+M_{g}^{I}+M_{g}^{J}+M_{g}^{K}\\ & & +4\kappa \left(L_{g}^{R}+L_{g}^{I}+L_{g}^{J}+L_{g}^{K}\right)]+\sum_{C_{kl}\in N_{u}\left(p,q\right)}{\bar{D}}_{pq}^{kl}\int_{0}^{+\infty }\left|K_{pq}\left(u\right)\right|\text{d}u\\ & & \times [M_{h}^{R}+M_{h}^{I}+M_{h}^{J}+M_{h}^{K}+4\kappa \left(L_{h}^{R}+L_{h}^{I}+L_{h}^{J}+L_{h}^{K}\right)], \end{array}$$

*system* (8) *has a unique almost periodic solution in* $Y^{*}=\left\{\varphi \in Y|∥\varphi ∥\leq \kappa \right\}$.

*proof*. Given φ∈Y, consider the following linear system

$$\begin{array}{ccc} X_{pq}^{\prime }\left(t\right) & = & -a_{pq}\left(t\right)X_{pq}\left(t\right)-\sum_{C_{kl}\in N_{r}\left(p,q\right)}B_{pq}^{kl}\left(t\right)F\left[t,\varphi \right]\varphi_{pq}\left(t\right)\\ & & -\sum_{C_{kl}\in N_{s}\left(p,q\right)}C_{pq}^{kl}\left(t\right)G\left[t,\varphi \right]\varphi_{pq}\left(t\right)+T_{pq}\left(t\right)\\ & & -\sum_{C_{kl}\in N_{u}\left(p,q\right)}D_{pq}^{kl}\left(t\right)\int_{0}^{+\infty }K_{pq}\left(t\right)H\left[t,u,\varphi \right]\text{d}u\varphi_{pq}\left(t\right),\;\;pq\in J. \end{array}$$ (15)

Together with (*A*_1_) and Lemma 2, the linear system

$$\begin{array}{c} X_{pq}^{\prime }\left(t\right)=-a_{pq}\left(t\right)X_{pq}\left(t\right),\;\;pq\in J \end{array}$$

admits an exponential dichotomy. By Lemma 1, we know that system (15) has a unique almost periodic solution which can be expressed as $X^{\varphi }\left(t\right)=\left\{X_{pq}^{\varphi }\left(t\right)\right\}$, where

$$\begin{array}{ccc} X_{pq}^{\varphi }\left(t\right) & = & \{\int_{-\infty }^{t}e^{-\int_{s}^{t}a_{pq}\left(u\right)\text{d}u}[-\sum_{C_{kl}\in N_{r}\left(p,q\right)}B_{pq}^{kl}\left(s\right)F\left[t,\varphi \right]\varphi_{pq}\left(s\right)\\ & & -\sum_{C_{kl}\in N_{s}\left(p,q\right)}C_{pq}^{kl}\left(s\right)G\left[t,\varphi \right]\varphi_{pq}\left(s\right)+T_{pq}\left(s\right)\\ & & -\sum_{C_{kl}\in N_{u}\left(p,q\right)}D_{pq}^{kl}\left(s\right)\int_{0}^{+\infty }K_{pq}\left(s\right)H\left[t,u,\varphi \right]\text{d}u\varphi_{pq}\left(s\right)]\text{d}s\},\;\;pq\in J. \end{array}$$

Now, we define a mapping: Γ:Y→Y with $\Gamma \left(\varphi \right)\left(t\right)=X^{\varphi }\left(t\right)=\left\{X_{pq}^{\varphi }\left(t\right)\right\}$, for ∀φ∈Y.

First, we will show that for any $\varphi \in Y^{*}$, $\Gamma \varphi \in Y^{*}$. Denote

$$\begin{array}{ccc} & & M_{pq}^{R}\left(s,\varphi \right)\\ & & \;=-\sum_{C_{kl}\in N_{r}\left(p,q\right)}B_{pq}^{kl}\left(s\right)(f^{R}\left[s,\varphi \right]\varphi_{pq}^{R}\left(s\right)-f^{I}\left[s,\varphi \right]\varphi_{pq}^{I}\left(s\right)-f^{J}\left[s,\varphi \right]\varphi_{pq}^{J}\left(s\right)\\ & & \;-f^{K}\left[s,\varphi \right]\varphi_{pq}^{K}\left(s\right))-\sum_{C_{kl}\in N_{s}\left(p,q\right)}C_{pq}^{kl}\left(s\right)(g^{R}\left[s,\varphi \right]\varphi_{pq}^{R}\left(s\right)-g^{I}\left[s,\varphi \right]\varphi_{pq}^{I}\left(s\right)\\ & & \;-g^{J}\left[s,\varphi \right]\varphi_{pq}^{J}\left(s\right)-g^{K}\left[s,\varphi \right]\varphi_{pq}^{K}\left(s\right))-\sum_{C_{kl}\in N_{u}\left(p,q\right)}D_{pq}^{kl}\left(s\right)(\int_{0}^{+\infty }K_{pq}\left(s\right)\\ & & \;\times h^{R}\left[s,u,\varphi \right]\text{d}u\varphi_{pq}^{R}\left(s\right)-\int_{0}^{+\infty }K_{pq}\left(s\right)h^{I}\left[s,u,\varphi \right]\text{d}u\varphi_{pq}^{I}\left(s\right)\\ & & \;-\int_{0}^{+\infty }K_{pq}\left(s\right)h^{J}\left[s,u,\varphi \right]\text{d}u\varphi_{pq}^{J}\left(s\right)-\int_{0}^{+\infty }K_{pq}\left(s\right)h^{K}\left[s,u,\varphi \right]\text{d}u\varphi_{pq}^{K}\left(s\right)),\;\;pq\in J. \end{array}$$

For pq∈J, we have

$$\begin{array}{ccc} & & |M_{pq}^{R}\left(s,\varphi \right)|\\ & & \;=|-\sum_{C_{kl}\in N_{r}\left(p,q\right)}B_{pq}^{kl}\left(s\right)(f^{R}\left[s,\varphi \right]\varphi_{pq}^{R}\left(s\right)-f^{I}\left[s,\varphi \right]\varphi_{pq}^{I}\left(s\right)-f^{J}\left[s,\varphi \right]\varphi_{pq}^{J}\left(s\right)\\ & & \;-f^{K}\left[s,\varphi \right]\varphi_{pq}^{K}\left(s\right))-\sum_{C_{kl}\in N_{s}\left(p,q\right)}C_{pq}^{kl}\left(s\right)(g^{R}\left[s,\varphi \right]\varphi_{pq}^{R}\left(s\right)-g^{I}\left[s,\varphi \right]\varphi_{pq}^{I}\left(s\right)\\ & & \;-g^{J}\left[s,\varphi \right]\varphi_{pq}^{J}\left(s\right)-g^{K}\left[s,\varphi \right]\varphi_{pq}^{K}\left(s\right))-\sum_{C_{kl}\in N_{u}\left(p,q\right)}D_{pq}^{kl}\left(s\right)(\int_{0}^{+\infty }K_{pq}\left(s\right)\\ & & \;\times h^{R}\left[s,u,\varphi \right]\text{d}u\varphi_{pq}^{R}\left(s\right)r-\int_{0}^{+\infty }K_{pq}\left(s\right)h^{I}\left[s,u,\varphi \right]\text{d}u\varphi_{pq}^{I}\left(s\right)\\ & & \;-\int_{0}^{+\infty }K_{pq}\left(s\right)h^{J}\left[s,u,\varphi \right]\text{d}u\varphi_{pq}^{J}\left(s\right)-\int_{0}^{+\infty }K_{pq}\left(s\right)h^{K}\left[s,u,\varphi \right]\text{d}u\varphi_{pq}^{K}\left(s\right))|\\ & & \;\leq \sum_{C_{kl}\in N_{r}\left(p,q\right)}{\bar{B}}_{pq}^{kl}\left(M_{f}^{R}+M_{f}^{I}+M_{f}^{J}+M_{f}^{K}\right){∥\varphi ∥}_{Y}+\sum_{C_{kl}\in N_{s}\left(p,q\right)}{\bar{C}}_{pq}^{kl}(M_{g}^{R}\\ & & \;+M_{g}^{I}+M_{g}^{J}+M_{g}^{K}){∥\varphi ∥}_{Y}+\sum_{C_{kl}\in N_{u}\left(p,q\right)}{\bar{D}}_{pq}^{kl}\int_{0}^{+\infty }K_{pq}\left(u\right)\text{d}u(M_{h}^{R}+M_{h}^{I}\\ & & \;+M_{h}^{J}+M_{h}^{K}){∥\varphi ∥}_{Y}\\ & & \;\leq \{\sum_{C_{kl}\in N_{r}\left(p,q\right)}{\bar{B}}_{pq}^{kl}\left(M_{f}^{R}+M_{f}^{I}+M_{f}^{J}+M_{f}^{K}\right)+\sum_{C_{kl}\in N_{s}\left(p,q\right)}{\bar{C}}_{pq}^{kl}(M_{g}^{R}+M_{g}^{I}\\ & & \;+M_{g}^{J}+M_{g}^{K})+\sum_{C_{kl}\in N_{u}\left(p,q\right)}{\bar{D}}_{pq}^{kl}\int_{0}^{+\infty }|K_{pq}\left(u\right)|\text{d}u(M_{h}^{R}+M_{h}^{I}+M_{h}^{J}\\ & & \;+M_{h}^{K})\}\kappa =\vartheta_{pq}\kappa , \end{array}$$

so we have

$$\begin{array}{ccc} |{\left(\Gamma \varphi \right)}_{pq}^{R}\left(t\right)| & \leq & |\int_{-\infty }^{t}e^{-\int_{s}^{t}a_{pq}\left(u\right)\text{d}u}\left(M_{pq}^{R}\left(s,\varphi \left(s\right)\right)+T_{pq}^{R}\left(s\right)\right)|\text{d}s\\ & \leq & \frac{\vartheta_{pq}\kappa +{\bar{T}}_{pq}^{R}}{{\underline{a}}_{pq}},\;\;pq\in J, \end{array}$$

repeat a similar calculation, we obtain

$$\begin{array}{ccc} |{\left(\Gamma \varphi \right)}_{pq}^{\nu }\left(t\right)| & \leq & \frac{\vartheta_{pq}\kappa +{\bar{T}}_{pq}^{\nu }}{{\underline{a}}_{pq}},\;\;pq\in J,\nu =I,J,K. \end{array}$$

Together with the above inequalities, we obtain

$$\begin{array}{ccc} {∥\Gamma \varphi ∥}_{Y} & \leq & \max_{pq\in J}\left\{\max_{\nu \in \Lambda }\left\{\frac{\vartheta_{pq}\kappa +{\bar{T}}_{pq}^{\nu }}{{\underline{a}}_{pq}}\right\}\right\}\leq \kappa , \end{array}$$

which following from (*A*_5_) implies that Γ is a self-mapping on $Y^{*}$.

Next, we show that Γ is a contraction mapping on $Y^{*}$. For any $\varphi ,\psi \in Y^{*}$, we have

$$\begin{array}{ccc} & & M_{pq}^{R}\left(s,\varphi \left(s\right)\right)-M_{pq}^{R}\left(s,\psi \left(s\right)\right)\\ & & \;=-\sum_{C_{kl}\in N_{r}\left(p,q\right)}B_{pq}^{kl}\left(s\right)[\left(f^{R}\left[s,\varphi \right]\varphi_{pq}^{R}\left(s\right)-f^{R}\left[s,\psi \right]\psi_{pq}^{R}\left(s\right)\right)-(f^{I}\left[s,\varphi \right]\varphi_{pq}^{I}\left(s\right)\\ & & \;-f^{I}\left[s,\psi \right]\psi_{pq}^{I}\left(s\right))-\left(f^{J}\left[s,\varphi \right]\varphi_{pq}^{J}\left(s\right)-f^{J}\left[s,\psi \right]\psi_{pq}^{J}\left(s\right)\right)-(f^{K}\left[s,\varphi \right]\varphi_{pq}^{K}\left(s\right)\\ & & \;-f^{K}\left[s,\psi \right]\psi_{pq}^{K}\left(s\right))]-\sum_{C_{kl}\in N_{s}\left(p,q\right)}C_{pq}^{kl}\left(s\right)[(g^{R}\left[s,\varphi \right]\varphi_{pq}^{R}\left(s\right)-g^{R}\left[s,\psi \right]\psi_{pq}^{R}\left(s\right)\\ & & \;-\left(g^{I}\left[s,\varphi \right]\varphi_{pq}^{I}\left(s\right)-g^{I}\left[s,\psi \right]\psi_{pq}^{I}\left(s\right)\right)-(g^{J}\left[s,\varphi \right]\psi_{pq}^{J}\left(s\right)-g^{J}\left[s,\psi \right]\\ & & \;\times \psi_{pq}^{J}\left(s\right)-\left(g^{K}\left[s,\varphi \right]\varphi_{pq}^{K}\left(s\right)-g^{K}\left[s,\psi \right]\psi_{pq}^{K}\left(s\right)\right)]-\sum_{C_{kl}\in N_{u}\left(p,q\right)}D_{pq}^{kl}\left(s\right)\\ & & \;\times [\int_{0}^{+\infty }K_{pq}\left(u\right)\left(h^{R}\left[s,u,\varphi \right]\varphi_{pq}^{R}\left(s\right)-h^{R}\left[s,u,\psi \right]\psi_{pq}^{R}\left(s\right)\right)\text{d}u\\ & & \;-\int_{0}^{+\infty }K_{pq}\left(u\right)\left(h^{I}\left[s,u,\varphi \right]\varphi_{pq}^{I}\left(s\right)-h^{I}\left[s,u,\psi \right]\psi_{pq}^{I}\left(s\right)\right)\text{d}u\\ & & \;-\int_{0}^{+\infty }K_{pq}\left(u\right)\left(h^{J}\left[s,u,\varphi \right]\varphi_{pq}^{J}\left(s\right)-h^{J}\left[s,u,\psi \right]\psi_{pq}^{J}\left(s\right)\right)\text{d}u\\ & & \;-\int_{0}^{+\infty }K_{pq}\left(u\right)\left(h^{K}\left[s,u,\varphi \right]\varphi_{pq}^{K}\left(s\right)-h^{K}\left[s,u,\psi \right]\psi_{pq}^{K}\left(s\right)\right)\text{d}u],\;pq\in J, \end{array}$$

so

$$\begin{array}{ccc} & & |M_{pq}^{R}\left(s,\varphi \right)-M_{pq}^{R}\left(s,\psi \right)|\\ & & \;\leq \sum_{C_{kl}\in N_{r}\left(p,q\right)}{\bar{B}}_{pq}^{kl}[M_{f}^{R}|\varphi_{pq}^{R}\left(s\right)-\psi_{pq}^{R}\left(s\right)|+M_{f}^{I}\left|\varphi_{pq}^{I}\left(s\right)-\psi_{pq}^{I}\left(s\right)\right|\\ & & \;+M_{f}^{J}|\varphi_{pq}^{J}\left(s\right)-\psi_{pq}^{J}\left(s\right)|+M_{f}^{K}|\varphi_{pq}^{K}\left(s\right)-\psi_{pq}^{K}\left(s\right)|+(L_{f}^{R}\left|\varphi_{pq}^{R}\left(s\right)-\psi_{pq}^{R}\left(s\right)\right|\\ & & \;+L_{f}^{I}|\varphi_{pq}^{I}\left(s\right)-\psi_{pq}^{I}\left(s\right)|+L_{f}^{J}|\varphi_{pq}^{J}\left(s\right)-\psi_{pq}^{J}\left(s\right)|+L_{f}^{K}|\varphi_{pq}^{K}\left(s\right)-\psi_{pq}^{K}\left(s\right)\left|\right)\\ & & \;\times \left(\right|\psi_{pq}^{R}\left(s\right)|+|\psi_{pq}^{I}\left(s\right)|+|\psi_{pq}^{J}\left(s\right)|+|\psi_{pq}^{K}\left(s\right)|)+\sum_{C_{kl}\in N_{s}\left(p,q\right)}{\bar{C}}_{pq}^{kl}\left[M_{g}^{R}\right|\varphi_{pq}^{R}\left(s\right)\\ & & \;-\psi_{pq}^{R}\left(s\right)|+M_{g}^{I}|\varphi_{pq}^{I}\left(s\right)-\psi_{pq}^{I}\left(s\right)|+M_{g}^{J}\left|\varphi_{pq}^{J}\left(s\right)-\psi_{pq}^{J}\left(s\right)\right|\\ & & \;+M_{g}^{K}|\varphi_{pq}^{K}\left(s\right)-\psi_{pq}^{K}\left(s\right)|+(L_{g}^{R}\left|\varphi_{pq}^{R}\left(s-\tau \left(s\right)\right)-\psi_{pq}^{R}\left(s-\tau \left(s\right)\right)\right|\\ & & \;+L_{g}^{I}|\varphi_{pq}^{I}\left(s-\tau \left(s\right)\right)-\psi_{pq}^{I}\left(s-\tau \left(s\right)\right)|+L_{g}^{J}\left|\varphi_{pq}^{J}\left(s-\tau \left(s\right)\right)-\psi_{pq}^{J}\left(s-\tau \left(s\right)\right)\right|\\ & & \;+L_{g}^{K}|\varphi_{pq}^{K}\left(s-\tau \left(s\right)\right)-\psi_{pq}^{K}\left(s-\tau \left(s\right)\right)|)(|\psi_{pq}^{R}\left(s\right)|+|\psi_{pq}^{I}\left(s\right)|+|\psi_{pq}^{J}\left(s\right)|\\ & & \;+|\psi_{pq}^{K}\left(s\right)|)]+\sum_{C_{kl}\in N_{u}\left(p,q\right)}{\bar{D}}_{pq}^{kl}[\int_{0}^{+\infty }K_{pq}\left(u\right)\text{d}u(M_{h}^{R}\left|\varphi_{pq}^{R}\left(s\right)-\psi_{pq}^{R}\left(s\right)\right|\\ & & \;+M_{h}^{I}|\varphi_{pq}^{I}\left(s\right)-\psi_{pq}^{I}\left(s\right)|+M_{h}^{J}|\varphi_{pq}^{J}\left(s\right)-\psi_{pq}^{J}\left(s\right)|+M_{h}^{K}|\varphi_{pq}^{K}\left(s\right)-\psi_{pq}^{K}\left(s\right)\left|\right)\\ & & \;+\int_{0}^{+\infty }K_{pq}\left(u\right)\text{d}u(L_{h}^{R}|\varphi_{pq}^{R}\left(s-u\right)-\psi_{pq}^{R}\left(s-u\right)\left|+L_{h}^{I}\right|\varphi_{pq}^{I}\left(s-u\right)\\ & & \;-\psi_{pq}^{I}\left(s-u\right))|+L_{h}^{J}|\varphi_{pq}^{J}\left(s-u\right)-\psi_{pq}^{J}\left(s-u\right)\left|+L_{h}^{K}\right|\varphi_{pq}^{K}\left(s-u\right)\\ & & \;-\psi_{pq}^{K}\left(s-u\right)|)\text{d}u(|\psi_{pq}^{R}\left(s\right)|+|\psi_{pq}^{I}\left(s\right)|+|\psi_{pq}^{J}\left(s\right)|+|\psi_{pq}^{K}\left(s\right)|)]\\ & & \;\leq \sum_{C_{kl}\in N_{r}\left(p,q\right)}{\bar{B}}_{pq}^{kl}[M_{f}^{R}+M_{f}^{I}+M_{f}^{J}+M_{f}^{K}+4\kappa (L_{f}^{R}+L_{f}^{I}+L_{f}^{J}\\ & & \;+L_{f}^{K})]∥\varphi -\psi ∥+\sum_{C_{kl}\in N_{s}\left(p,q\right)}{\bar{C}}_{pq}^{kl}[M_{g}^{R}+M_{g}^{I}+M_{g}^{J}+M_{g}^{K}+4\kappa (L_{g}^{R}+L_{g}^{I}\\ & & \;+L_{g}^{J}+L_{g}^{K})]∥\varphi -\psi ∥+\sum_{C_{kl}\in N_{u}\left(p,q\right)}{\bar{D}}_{pq}^{kl}\int_{0}^{\infty }|K_{pq}\left(u\right)|\text{d}u[M_{h}^{R}+M_{h}^{I}+M_{h}^{J}\\ & & \;+M_{h}^{K}+4\kappa \left(L_{h}^{R}+L_{h}^{I}+L_{h}^{J}+L_{h}^{K}\right)]∥\varphi -\psi ∥=\mu_{pq}∥\varphi -\psi ∥,\;\;pq\in J. \end{array}$$

Hence, we have

$$\begin{array}{ccc} |{\left(\Gamma \varphi \right)}^{R}-{\left(\Gamma \psi \right)}^{R}| & = & |\int_{-\infty }^{t}e^{-\int_{s}^{t}a_{pq}\left(u\right)\text{d}u}\left[M^{R}\left(s,\varphi \left(s\right)\right)-M^{R}\left(s,\psi \left(s\right)\right)\right]\text{d}s|\\ & = & \frac{\mu_{pq}}{{\underline{a}}_{pq}}{∥\varphi -\psi ∥}_{Y},\;\;pq\in J. \end{array}$$

Similarly, one can obtain

$$\begin{array}{c} |{\left(\Gamma \varphi \right)}^{\nu }-{\left(\Gamma \psi \right)}^{\nu }|\leq \frac{\mu_{pq}}{{\underline{a}}_{pq}}{∥\varphi -\psi ∥}_{Y},\;\;pq\in J,\nu =I,J,K. \end{array}$$

Therefore,

$$\begin{array}{c} ∥\Gamma \varphi -\Gamma \psi ∥\leq \mu ∥\varphi -\psi ∥, \end{array}$$

which implies that Γ is a contraction mapping. According to the Banach fixed point theorem, Γ has a unique fixed point in $Y^{*}$, which means that system (8) has a unique almost periodic solution in $Y^{*}$. The proof is complete.

**Theorem 2**. *Assume* (*A*_1_)-(*A*_4_) *hold and suppose further that*

(*A*_5_) *There exists a positive constant* λ *such that*
  $$\begin{array}{c} \gamma =\max_{pq\in J}\left\{\gamma_{pq}\right\}<0, \end{array}$$
  *where*
  $$\begin{array}{ccc} \gamma_{pq} & = & \left(\lambda -\left({\underline{a}}_{pq}+{\underline{d}}_{pq}\right)\right)+\sum_{C_{kl}\in N_{r}\left(p,q\right)}{\bar{B}}_{pq}^{kl}[M_{f}^{R}+M_{f}^{I}+M_{f}^{J}+M_{f}^{K}\\ & & +4\kappa \left(L_{f}^{R}+L_{f}^{I}+L_{f}^{J}+L_{f}^{K}\right)]+\sum_{C_{kl}\in N_{s}\left(p,q\right)}{\bar{C}}_{pq}^{kl}[M_{g}^{R}+M_{g}^{I}\\ & & +M_{g}^{J}+M_{g}^{K}+\frac{4\kappa e^{\lambda \bar{\tau }}}{1-\alpha }\left(L_{g}^{R}+L_{g}^{I}+L_{g}^{J}+L_{g}^{K}\right)]\\ & & +\sum_{C_{kl}\in N_{u}\left(p,q\right)}{\bar{D}}_{pq}^{kl}\int_{0}^{+\infty }|K_{pq}\left(u\right)|[M_{h}^{R}+M_{h}^{I}+M_{h}^{J}+M_{h}^{K}\\ & & +4\kappa e^{\lambda u}\left(L_{h}^{R}+L_{h}^{I}+L_{h}^{J}+L_{h}^{K}\right)]\text{d}u+\sum_{C_{kl}\in N_{v}\left(p,q\right)}{\bar{E}}_{pq}^{kl}[M_{w}^{R}\\ & & +M_{w}^{I}+M_{w}^{J}+M_{w}^{K}+\frac{4\kappa e^{\lambda \bar{\delta }}}{1-\beta }\left(L_{w}^{R}+L_{w}^{I}+L_{w}^{J}+L_{w}^{K}\right)]. \end{array}$$

*Then the drive system* (1) *and response system* (9) *are globally exponentially synchronized*.

*proof*. Let us construct a Lyapunov function *V*(*t*) as follows

$$\begin{array}{c} V\left(t\right)=V^{R}\left(t\right)+V^{I}\left(t\right)+V^{J}\left(t\right)+V^{K}\left(t\right), \end{array}$$

where $V^{\nu }\left(t\right)=\sum_{pq\in J}\left(\right|z_{pq}^{\nu }\left(t\right)\left|e^{\lambda t}+\Theta_{pq}\right)$, pq∈J, *ν* ∈ Λ and

$$\begin{array}{ccc} \Theta_{pq}\left(t\right) & = & 4\kappa [\sum_{C_{kl}\in N_{r}\left(p,q\right)}{\bar{C}}_{pq}^{kl}\frac{e^{\lambda \bar{\tau }}}{1-\alpha }\int_{t-\tau (t)}^{t}(L_{g}^{R}|z_{kl}^{R}\left(s\right)|+L_{g}^{I}\left|z_{kl}^{I}\left(s\right)\right|\\ & & +L_{g}^{J}|z_{kl}^{J}\left(s\right)|+L_{g}^{K}|z_{kl}^{K}\left(s\right)\left|\right)e^{\lambda s}\text{d}s+\sum_{C_{kl}\in N_{u}\left(p,q\right)}{\bar{D}}_{pq}^{kl}\int_{0}^{+\infty }K_{pq}\left(u\right)\\ & & \times e^{\lambda u}\int_{t-u}^{t}(L_{h}^{R}|z_{kl}^{R}\left(s\right)|+L_{h}^{I}|z_{kl}^{I}\left(s\right)|+L_{h}^{J}|z_{kl}^{J}\left(s\right)|+L_{h}^{K}|z_{kl}^{K}\left(s\right)\left|\right)e^{\lambda s}\text{d}s\text{d}u\\ & & +\sum_{C_{kl}\in N_{v}\left(p,q\right)}{\bar{E}}_{pq}^{kl}\frac{e^{\lambda \bar{\delta }}}{1-\beta }\int_{t-\delta (t)}^{t}(L_{w}^{R}|z_{kl}^{R}\left(s\right)|+L_{w}^{I}|z_{kl}^{I}\left(s\right)|+L_{w}^{J}\left|z_{kl}^{J}\left(s\right)\right|\\ & & +L_{w}^{K}|z_{kl}^{K}\left(s\right)|)e^{\lambda s}\text{d}s]. \end{array}$$

From (11)–(14), for any *t* > 0, *ν* ∈ Λ, pq∈J, we have

$$\begin{array}{ccc} & & D^{+}\left|z^{\nu }\left(t\right)\right|\\ & & \;\leq -\left({\underline{a}}_{pq}+{\underline{d}}_{pq}\right)|z_{pq}^{\nu }|+\sum_{C_{kl}\in N_{r}\left(p,q\right)}{\bar{B}}_{pq}^{kl}[M_{f}^{R}|z_{pq}^{R}\left(t\right)|+M_{f}^{I}|z_{pq}^{I}\left(t\right)|+M_{f}^{J}\left|z_{pq}^{J}\left(t\right)\right|\\ & & \;+M_{f}^{K}|z_{pq}^{K}\left(t\right)|+4\kappa (L_{f}^{R}|z_{kl}^{R}\left(t\right)|+L_{f}^{I}|z_{kl}^{I}\left(t\right)|+L_{f}^{J}|z_{kl}^{J}\left(t\right)|+L_{f}^{K}|z_{kl}^{K}\left(t\right)\left|\right)\\ & & \;+\sum_{C_{kl}\in N_{s}\left(p,q\right)}{\bar{C}}_{pq}^{kl}[M_{g}^{R}|z_{pq}^{R}\left(t\right)|+M_{g}^{I}|z_{pq}^{I}\left(t\right)|+M_{g}^{J}|z_{pq}^{J}\left(t\right)|+M_{g}^{K}\left|z_{pq}^{K}\left(t\right)\right|\\ & & \;+4\kappa (L_{g}^{R}|z_{kl}^{R}\left(t-\tau \left(t\right)\right)|+L_{g}^{I}|z_{kl}^{I}\left(t-\tau \left(t\right)\right)|+L_{g}^{J}|z_{kl}^{J}\left(t-\tau \left(t\right)\right)|\\ & & \;+L_{g}^{K}|z_{kl}^{K}\left(t-\tau \left(t\right)\right)|)]+\sum_{C_{kl}\in N_{u}\left(p,q\right)}{\bar{D}}_{pq}^{kl}\int_{0}^{+\infty }K_{pq}\left(u\right)[M_{h}^{R}\left|z_{pq}^{R}\left(t\right)\right|\\ & & \;+M_{h}^{I}|z_{pq}^{I}\left(t\right)|+M_{h}^{J}|z_{pq}^{J}\left(t\right)|+M_{h}^{K}|z_{pq}^{K}\left(t\right)|+4\kappa (L_{h}^{R}\left|z_{kl}^{R}\left(t-u\right)\right|\\ & & \;+L_{h}^{I}|z_{kl}^{I}\left(t-u\right)|+L_{h}^{J}|z_{kl}^{J}\left(t-u\right)|+L_{h}^{K}|z_{kl}^{K}\left(t-u\right)|)]\text{d}u\\ & & \;+\sum_{C_{kl}\in N_{v}\left(p,q\right)}{\bar{E}}_{pq}^{kl}[M_{w}^{R}|z_{pq}^{R}\left(t\right)|+M_{w}^{I}|z_{pq}^{I}\left(t\right)|+M_{w}^{J}|z_{pq}^{J}\left(t\right)|+M_{w}^{K}\left|z_{pq}^{K}\left(t\right)\right|\\ & & \;+4\kappa (L_{w}^{R}|z_{kl}^{R}\left(t-\delta \left(t\right)\right)|+L_{w}^{I}|z_{kl}^{I}\left(t-\delta \left(t\right)\right))|+L_{w}^{J}\left|z_{kl}^{J}\left(t-\delta \left(t\right)\right)\right|\\ & & \;+L_{w}^{K}|z_{kl}^{K}\left(t-\delta \left(t\right)\right)|)]. \end{array}$$

Computing the derivative of *V*_1_(*t*) along the solution of the error system (10), we obtain

$$\begin{array}{ccc} & & D^{+}V^{R}\left(t\right)\\ & & \;=\sum_{pq\in J}\{\lambda e^{\lambda t}|z_{pq}^{R}\left(t\right)\left|+sign\left(z_{pq}^{R}\left(t\right)\right)D^{+}\left(z_{pq}^{R}\left(t\right)\right)e^{\lambda t}+D^{+}\Theta_{pq}\left(t\right)\right\}\\ & & \;\leq \sum_{pq\in J}\left\{(\lambda -\left({\underline{a}}_{pq}+{\underline{d}}_{pq}\right)\right)e^{\lambda t}|z_{pq}^{R}|+e^{\lambda t}\sum_{C_{kl}\in N_{r}\left(p,q\right)}{\bar{B}}_{pq}^{kl}[M_{f}^{R}\left|z_{pq}^{R}\left(t\right)\right|\\ & & \;+M_{f}^{I}|z_{pq}^{I}\left(t\right)|+M_{f}^{J}|z_{pq}^{J}\left(t\right)|+M_{f}^{K}|z_{pq}^{K}\left(t\right)|+4\kappa (L_{f}^{R}|z_{kl}^{R}\left(t\right)|+L_{f}^{I}\left|z_{kl}^{I}\left(t\right)\right|\\ & & \;+L_{f}^{J}|z_{kl}^{J}\left(t\right)|+L_{f}^{K}|z_{kl}^{K}\left(t\right)|)]+e^{\lambda t}\sum_{C_{kl}\in N_{s}\left(p,q\right)}{\bar{C}}_{pq}^{kl}[M_{g}^{R}\left|z_{pq}^{R}\left(t\right)\right|\\ & & \;+M_{g}^{I}|z_{pq}^{I}\left(t\right)|+M_{g}^{J}|z_{pq}^{J}\left(t\right)|+M_{g}^{K}|z_{pq}^{K}\left(t\right)|+4\kappa (L_{g}^{R}\left|z_{kl}^{R}\left(t-\tau \left(t\right)\right)\right|\\ & & \;+L_{g}^{I}|z_{kl}^{I}\left(t-\tau \left(t\right)\right)|+L_{g}^{J}|z_{kl}^{J}\left(t-\tau \left(t\right)\right)|+L_{g}^{K}|z_{kl}^{K}\left(t-\tau \left(t\right)\right)|)]\\ & & \;+e^{\lambda t}\sum_{C_{kl}\in N_{u}\left(p,q\right)}{\bar{D}}_{pq}^{kl}\int_{0}^{+\infty }K_{pq}\left(u\right)[(M_{h}^{R}|z_{pq}^{R}\left(t\right)|+M_{h}^{I}\left|z_{pq}^{I}\left(t\right)\right|\\ & & \;+M_{h}^{J}|z_{pq}^{J}\left(t\right)|+M_{h}^{K}|z_{pq}^{K}\left(t\right)|)+4\kappa (L_{h}^{R}|z_{kl}^{R}\left(t-u\right)|+L_{h}^{I}\left|z_{kl}^{I}\left(t-u\right)\right|\\ & & \;+L_{h}^{J}|z_{kl}^{J}\left(t-u\right)|+L_{h}^{K}|z_{kl}^{K}\left(t-u\right)|)]\text{d}u+e^{\lambda t}\sum_{C_{kl}\in N_{v}\left(p,q\right)}{\bar{E}}_{pq}^{kl}\\ & & \;\times [M_{w}^{R}|z_{pq}^{R}\left(t\right)|+M_{w}^{I}|z_{pq}^{I}\left(t\right)|+M_{w}^{J}|z_{pq}^{J}\left(t\right)|+M_{w}^{K}|z_{pq}^{K}\left(t\right)|\\ & & \;+4\kappa (L_{w}^{R}|z_{kl}^{R}\left(t-\delta \left(t\right)\right)|+L_{w}^{I}|z_{kl}^{I}\left(t-\delta \left(t\right)\right))|+L_{w}^{J}\left|z_{kl}^{J}\left(t-\delta \left(t\right)\right)\right|\\ & & \;+L_{w}^{K}|z_{kl}^{K}\left(s-\delta \left(t\right)\right)|)]+\sum_{C_{kl}\in N_{r}\left(p,q\right)}4\kappa {\bar{C}}_{pq}^{kl}\frac{e^{\lambda \bar{\tau }}}{1-\alpha }\\ & & \;\times (L_{g}^{R}|z_{kl}^{R}\left(t\right)|+L_{g}^{I}|z_{kl}^{I}\left(t\right)|+L_{g}^{J}|z_{kl}^{J}\left(t\right)|+L_{g}^{K}|z_{kl}^{K}\left(t\right)\left|\right)e^{\lambda t}\\ & & \;-\sum_{C_{kl}\in N_{r}\left(p,q\right)}4\kappa {\bar{C}}_{pq}^{kl}\frac{e^{\lambda \bar{\tau }}}{1-\alpha }(L_{g}^{R}|z_{kl}^{R}\left(t-\tau \left(t\right)\right)|+L_{g}^{I}\left|z_{kl}^{I}\left(t-\tau \left(t\right)\right)\right|\\ & & \;+L_{g}^{J}|z_{kl}^{J}\left(t-\tau \left(t\right)\right)|+L_{g}^{K}|z_{kl}^{K}\left(t-\tau \left(t\right)\right)\left|\right)e^{\lambda (t-\tau (t))}\left(1-\tau^{\prime }\left(t\right)\right)\\ & & \;+\sum_{C_{kl}\in N_{u}\left(p,q\right)}4\kappa {\bar{D}}_{pq}^{kl}\int_{0}^{+\infty }K_{pq}\left(u\right)e^{\lambda u}(L_{h}^{R}|z_{kl}^{R}\left(t\right)|+L_{h}^{I}\left|z_{kl}^{I}\left(t\right)\right|\\ & & \;+L_{h}^{J}|z_{kl}^{J}\left(t\right)|+L_{h}^{K}|z_{kl}^{K}\left(t\right)\left|\right)e^{\lambda t}\text{d}u-\sum_{C_{kl}\in N_{u}\left(p,q\right)}4\kappa {\bar{D}}_{pq}^{kl}\\ & & \;\times \int_{0}^{+\infty }K_{pq}\left(u\right)e^{\lambda u}(L_{h}^{R}|z_{kl}^{R}\left(t-u\right)|+L_{h}^{I}|z_{kl}^{I}\left(t-u\right)|+L_{h}^{J}\left|z_{kl}^{J}\left(t-u\right)\right|\\ & & \;+L_{h}^{K}|z_{kl}^{K}\left(t-u\right)|)e^{\lambda (t-u)}\text{d}u+\sum_{C_{kl}\in N_{v}\left(p,q\right)}4\kappa {\bar{E}}_{pq}^{kl}\frac{e^{\lambda \bar{\delta }}}{1-\beta }(L_{w}^{R}\left|z_{kl}^{R}\left(t\right)\right|+\\ & & \;L_{w}^{I}|z_{kl}^{I}\left(t\right)|+L_{w}^{J}|z_{kl}^{J}\left(t\right)|+L_{w}^{K}|z_{kl}^{K}\left(t\right)\left|\right)e^{\lambda t}-\sum_{C_{kl}\in N_{v}\left(p,q\right)}4\kappa {\bar{E}}_{pq}^{kl}\frac{e^{\lambda \bar{\delta }}}{1-\beta }\\ & & \;\times (L_{w}^{R}|z_{kl}^{R}\left(t-\delta \left(t\right)\right)|+L_{w}^{I}|z_{kl}^{I}\left(t-\delta \left(t\right)\right)|+L_{w}^{J}|z_{kl}^{J}\left(t-\delta \left(t\right)\right)|\\ & & \;+L_{w}^{K}|z_{kl}^{K}\left(t-\delta \left(t\right)\right)|)e^{\lambda (t-\delta (t))}\left(1-\delta^{\prime }\left(t\right)\right)]\}\\ & & \;\leq \sum_{pq\in J}\left\{(\lambda -\left({\underline{a}}_{pq}+{\underline{d}}_{pq}\right)\right)e^{\lambda t}|z_{pq}^{R}|+e^{\lambda t}\sum_{C_{kl}\in N_{r}\left(p,q\right)}{\bar{B}}_{pq}^{kl}[M_{f}^{R}\left|z_{pq}^{R}\left(t\right)\right|\\ & & \;+M_{f}^{I}|z_{pq}^{I}\left(t\right)|+M_{f}^{J}|z_{pq}^{J}\left(t\right)|+M_{f}^{K}|z_{pq}^{K}\left(t\right)|+4\kappa (L_{f}^{R}|z_{kl}^{R}\left(t\right)|+L_{f}^{I}\left|z_{kl}^{I}\left(t\right)\right|\\ & & \;+L_{f}^{J}|z_{kl}^{J}\left(t\right)|+L_{f}^{K}|z_{kl}^{K}\left(t\right)|)]+e^{\lambda t}\sum_{C_{kl}\in N_{s}\left(p,q\right)}{\bar{C}}_{pq}^{kl}[M_{g}^{R}\left|z_{pq}^{R}\left(t\right)\right|\\ & & \;+M_{g}^{I}|z_{pq}^{I}\left(t\right)|+M_{g}^{J}|z_{pq}^{J}\left(t\right)|+M_{g}^{K}|z_{pq}^{K}\left(t\right)|+4\kappa (L_{g}^{R}\left|z_{kl}^{R}\left(t-\tau \left(t\right)\right)\right|\\ & & \;+L_{g}^{I}|z_{kl}^{I}\left(t-\tau \left(t\right)\right)|+L_{g}^{J}|z_{kl}^{J}\left(t-\tau \left(t\right)\right)|+L_{g}^{K}|z_{kl}^{K}\left(t-\tau \left(t\right)\right)|)]\\ & & \;+e^{\lambda t}\sum_{C_{kl}\in N_{u}\left(p,q\right)}{\bar{D}}_{pq}^{kl}\int_{0}^{+\infty }K_{pq}\left(u\right)[(M_{h}^{R}|z_{pq}^{R}\left(t\right)|+M_{h}^{I}\left|z_{pq}^{I}\left(t\right)\right|\\ & & \;+M_{h}^{J}|z_{pq}^{J}\left(t\right)|+M_{h}^{K}|z_{pq}^{K}\left(t\right)|)+4\kappa (L_{h}^{R}|z_{kl}^{R}\left(t-u\right)|+L_{h}^{I}\left|z_{kl}^{I}\left(t-u\right)\right|\\ & & \;+L_{h}^{J}\times |z_{kl}^{J}\left(t-u\right)|+L_{h}^{K}|z_{kl}^{K}\left(t-u\right)|)]\text{d}u+e^{\lambda t}\sum_{C_{kl}\in N_{v}\left(p,q\right)}{\bar{E}}_{pq}^{kl}\\ & & \;\times [M_{w}^{R}|z_{pq}^{R}\left(t\right)|+M_{w}^{I}|z_{pq}^{I}\left(t\right)|+M_{w}^{J}|z_{pq}^{J}\left(t\right)|+M_{w}^{K}|z_{pq}^{K}\left(t\right)|\\ & & \;+4\kappa (L_{w}^{R}|z_{kl}^{R}\left(t-\delta \left(t\right)\right)|+L_{w}^{I}|z_{kl}^{I}\left(t-\delta \left(t\right)\right))|+L_{w}^{J}\left|z_{kl}^{J}\left(t-\delta \left(t\right)\right)\right|\\ & & \;+L_{w}^{K}|z_{kl}^{K}\left(s-\delta \left(t\right)\right)|)]+\sum_{C_{kl}\in N_{r}\left(p,q\right)}4\kappa {\bar{C}}_{pq}^{kl}\frac{e^{\lambda \bar{\tau }}}{1-\alpha }(L_{g}^{R}|z_{kl}^{R}\left(t\right)|+L_{g}^{I}\left|z_{kl}^{I}\left(t\right)\right|\\ & & \;+L_{g}^{J}|z_{kl}^{J}\left(t\right)|+L_{g}^{K}|z_{kl}^{K}\left(t\right)|)e^{\lambda t}-4\kappa \sum_{C_{kl}\in N_{r}\left(p,q\right)}{\bar{C}}_{pq}^{kl}(L_{g}^{R}\left|z_{kl}^{R}\left(t-\tau \left(t\right)\right)\right|\\ & & \;+L_{g}^{I}|z_{kl}^{I}\left(t-\tau \left(t\right)\right)|+L_{g}^{J}\left|z_{kl}^{J}\left(t-\tau \left(t\right)\right)\right|\\ & & \;+L_{g}^{K}|z_{kl}^{K}\left(t-\tau \left(t\right)\right)|)e^{\lambda t}+4\kappa \sum_{C_{kl}\in N_{u}\left(p,q\right)}{\bar{D}}_{pq}^{kl}\int_{0}^{+\infty }\left|K_{pq}\left(u\right)\right|e^{\lambda u}\\ & & \;(L_{h}^{R}|z_{kl}^{R}\left(t\right)|+L_{h}^{I}|z_{kl}^{I}\left(t\right)|+L_{h}^{J}|z_{kl}^{J}\left(t\right)|+L_{h}^{K}|z_{kl}^{K}\left(t\right)\left|\right)e^{\lambda t}\text{d}u\\ & & \;-4\kappa \sum_{C_{kl}\in N_{u}\left(p,q\right)}{\bar{D}}_{pq}^{kl}\int_{0}^{+\infty }|K_{pq}\left(u\right)|e^{\lambda u}(L_{h}^{R}|z_{kl}^{R}\left(t-u\right)|+L_{h}^{I}\left|z_{kl}^{I}\left(t-u\right)\right|\\ & & \;+L_{h}^{J}|z_{kl}^{J}\left(t-u\right)|+L_{h}^{K}|z_{kl}^{K}\left(t-u\right)\left|\right)e^{\lambda (t-u)}\text{d}u+\sum_{C_{kl}\in N_{v}\left(p,q\right)}4\kappa {\bar{E}}_{pq}^{kl}\frac{e^{\lambda \bar{\delta }}}{1-\beta }\\ & & \;\times (L_{w}^{R}|z_{kl}^{R}\left(t\right)|+L_{w}^{I}|z_{kl}^{I}\left(t\right)|+L_{w}^{J}|z_{kl}^{J}\left(t\right)|+L_{w}^{K}|z_{kl}^{K}\left(t\right)\left|\right)e^{\lambda t}\\ & & \;-4\kappa \sum_{C_{kl}\in N_{v}\left(p,q\right)}{\bar{E}}_{pq}^{kl}(L_{w}^{R}|z_{kl}^{R}\left(t-\delta \left(t\right)\right)|+L_{w}^{I}\left|z_{kl}^{I}\left(t-\delta \left(t\right)\right)\right|\\ & & \;+L_{w}^{J}|z_{kl}^{J}\left(t-\delta \left(t\right)\right)|+L_{w}^{K}|z_{kl}^{K}\left(t-\delta \left(t\right)\right)|)e^{\lambda t}]\}\\ & & \;\leq e^{\lambda t}\sum_{pq\in J}\{\left(\lambda -\left({\underline{a}}_{pq}+{\underline{d}}_{pq}\right)\right)+\sum_{C_{kl}\in N_{r}\left(p,q\right)}{\bar{B}}_{pq}^{kl}[M_{f}^{R}+M_{f}^{I}+M_{f}^{J}\\ & & \;+M_{f}^{K}+4\kappa \left(L_{f}^{R}+L_{f}^{I}+L_{f}^{J}+L_{f}^{K}\right)]+\sum_{C_{kl}\in N_{s}\left(p,q\right)}{\bar{C}}_{pq}^{kl}[M_{g}^{R}+M_{g}^{I}\\ & & \;+M_{g}^{J}+M_{g}^{K}+\frac{4\kappa e^{\lambda \bar{\tau }}}{1-\alpha }\left(L_{g}^{R}\right]+L_{g}^{I}+L_{g}^{J}+L_{g}^{K})\\ & & \;+\sum_{C_{kl}\in N_{u}\left(p,q\right)}{\bar{D}}_{pq}^{kl}\int_{0}^{+\infty }|K_{pq}\left(u\right)|[M_{h}^{R}+M_{h}^{I}+M_{h}^{J}+M_{h}^{K}\\ & & \;+4\kappa e^{\lambda u}\left(L_{h}^{R}+L_{h}^{I}+L_{h}^{J}+L_{h}^{K}\right)]\text{d}u+\sum_{C_{kl}\in N_{v}\left(p,q\right)}{\bar{E}}_{pq}^{kl}[M_{w}^{R}+M_{w}^{I}\\ & & \;+M_{w}^{J}+M_{w}^{K}+\frac{4\kappa e^{\lambda \bar{\delta }}}{1-\beta }\left(L_{w}^{R}+L_{w}^{I}+L_{w}^{J}+L_{w}^{K}\right)]\}∥z∥. \end{array}$$ (16)

Repeat a similar calculation, we obtain

$$\begin{array}{ccc} & & D^{+}V^{\nu }\left(t\right)\\ & & \;\leq e^{\lambda t}\sum_{pq\in J}\{\left(\lambda -\left({\underline{a}}_{pq}+{\underline{d}}_{pq}\right)\right)+\sum_{C_{kl}\in N_{r}\left(p,q\right)}{\bar{B}}_{pq}^{kl}[M_{f}^{R}+M_{f}^{I}+M_{f}^{J}\\ & & \;+M_{f}^{K}+4\kappa \left(L_{f}^{R}+L_{f}^{I}+L_{f}^{J}+L_{f}^{K}\right)]+\sum_{C_{kl}\in N_{s}\left(p,q\right)}{\bar{C}}_{pq}^{kl}[M_{g}^{R}+M_{g}^{I}\\ & & \;+M_{g}^{J}+M_{g}^{K}+\frac{4\kappa e^{\lambda \bar{\tau }}}{1-\alpha }\left(L_{g}^{R}\right)]+L_{g}^{I}+L_{g}^{J}+L_{g}^{K}\\ & & \;+\sum_{C_{kl}\in N_{u}\left(p,q\right)}{\bar{D}}_{pq}^{kl}\int_{0}^{+\infty }|K_{pq}\left(u\right)|[M_{h}^{R}+M_{h}^{I}+M_{h}^{J}+M_{h}^{K}\\ & & \;+4\kappa e^{\lambda u}\left(L_{h}^{R}+L_{h}^{I}+L_{h}^{J}+L_{h}^{K}\right)]\text{d}u+\sum_{C_{kl}\in N_{v}\left(p,q\right)}{\bar{E}}_{pq}^{kl}[M_{w}^{R}+M_{w}^{I}\\ & & \;+M_{w}^{J}+M_{w}^{K}+\frac{4\kappa e^{\lambda \bar{\delta }}}{1-\beta }\left(L_{w}^{R}+L_{w}^{I}+L_{w}^{J}+L_{w}^{K}\right)]\}∥z∥,\;\;\nu =I,J,K. \end{array}$$ (17)

It follows from (*A*_5_), (16) and (17) that

$$\begin{array}{c} D^{+}V\left(t\right)\leq 0, \end{array}$$

which implies that *V*(*t*) ≤ *V*(0) for all *t* ≥ 0.

On the other hand, we have

$$\begin{array}{ccc} V(0) & \leq & \sum_{pq\in J}\{1+\frac{4\kappa (e^{\lambda \bar{\tau }}-1)}{\lambda (1-\alpha )}\sum_{C_{kl}\in N_{s}\left(p,q\right)}{\bar{C}}_{pq}^{kl}\left[L_{g}^{R}+L_{g}^{I}+L_{g}^{J}+L_{g}^{K}\right]\\ & & +\sum_{C_{kl}\in N_{u}\left(p,q\right)}4\kappa {\bar{D}}_{pq}^{kl}[\int_{0}^{+\infty }|K_{pq}\left(u\right)\left|\frac{e^{\lambda u}-1}{\lambda }\text{d}u\left(L_{h}^{R}+L_{h}^{I}+L_{h}^{J}+L_{h}^{K}\right)\right]\\ & & +\frac{4\kappa (e^{\lambda \bar{\delta }}-1)}{\lambda (1-\beta )}\sum_{C_{kl}\in N_{v}\left(p,q\right)}{\bar{E}}_{pq}^{kl}\left[L_{w}^{R}+L_{w}^{I}+L_{w}^{J}+L_{w}^{K}\right)\}∥\psi -\varphi ∥. \end{array}$$

We also have

$$\begin{array}{c} {∥y(t)-x(t)∥}_{0}\leq V\left(t\right)e^{\lambda t}\leq V\left(0\right)e^{\lambda t}\leq M∥\psi -\varphi ∥e^{-\lambda t},\;t\geq 0, \end{array}$$

where

$$\begin{array}{ccc} M & = & \sum_{pq\in J}\{1+\frac{4\kappa (e^{\lambda \bar{\tau }}-1)}{\lambda (1-\alpha )}\sum_{C_{kl}\in N_{s}\left(p,q\right)}{\bar{C}}_{pq}^{kl}\left[L_{g}^{R}+L_{g}^{I}+L_{g}^{J}+L_{g}^{K}\right]\\ & & +\sum_{C_{kl}\in N_{u}\left(p,q\right)}4\kappa {\bar{D}}_{pq}^{kl}[\int_{0}^{+\infty }|K_{pq}\left(u\right)\left|\frac{e^{\lambda u}-1}{\lambda }\text{d}u\left(L_{h}^{R}+L_{h}^{I}+L_{h}^{J}+L_{h}^{K}\right)\right]\\ & & +\frac{4\kappa (e^{\lambda \bar{\delta }}-1)}{\lambda (1-\beta )}\sum_{C_{kl}\in N_{v}\left(p,q\right)}{\bar{E}}_{pq}^{kl}\left[L_{w}^{R}+L_{w}^{I}+L_{w}^{J}+L_{w}^{K})\right\}>0. \end{array}$$

Therefore, the drive system (1) and the response system (9) are globally exponentially synchronized. The proof is complete.

## A numerical example

In this section, an example is shown for the effectiveness of the proposed method in this paper.

**Example 1**. *If the following QVSICNN as the drive system*:

$$\begin{array}{ccc} x_{pq}^{\prime }\left(t\right) & = & -a_{pq}\left(t\right)x_{pq}\left(t\right)-\sum_{C_{kl}\in N_{r}\left(p,q\right)}B_{pq}^{kl}\left(t\right)f\left(x_{kl}\left(t\right)\right)x_{pq}\left(t\right)\\ & & -\sum_{C_{kl}\in N_{s}\left(p,q\right)}C_{pq}^{kl}\left(t\right)g\left(x_{kl}\left(t-\tau \left(t\right)\right)\right)x_{pq}\left(t\right)+T_{pq}\left(t\right)\\ & & -\sum_{C_{kl}\in N_{u}\left(p,q\right)}D_{pq}^{kl}\left(t\right)\int_{0}^{+\infty }K_{pq}\left(u\right)h\left(x_{kl}\left(t-u\right)\right)\text{d}ux_{pq}\left(t\right) \end{array}$$ (18)

*and the corresponding response system is defined as*

$$\begin{array}{ccc} y_{pq}^{\prime }\left(t\right) & = & -a_{pq}\left(t\right)y_{pq}\left(t\right)-\sum_{C_{kl}\in N_{r}\left(p,q\right)}B_{pq}^{kl}\left(t\right)f\left(y_{kl}\left(t\right)\right)y_{pq}\left(t\right)\\ & & -\sum_{C_{kl}\in N_{s}\left(p,q\right)}C_{pq}^{kl}\left(t\right)g\left(y_{kl}\left(t-\tau \left(t\right)\right)\right)y_{pq}\left(t\right)+T_{pq}\left(t\right)+U_{pq}\left(t\right)\\ & & -\sum_{C_{kl}\in N_{u}\left(p,q\right)}D_{pq}^{kl}\left(t\right)\int_{0}^{+\infty }K_{pq}\left(u\right)h\left(y_{kl}\left(t-u\right)\right)\text{d}uy_{pq}\left(t\right), \end{array}$$ (19)

*where* *K*_*pq*_(*u*) = (cos *u*)*e*^−2*u*^, *p*, *q* = 1, 2, *r* = *s* = *u* = *v* = 1, $\tau \left(t\right)=\frac{1}{2}\sin t$, $\delta \left(t\right)=\frac{1}{3}\sin t$, *and the coefficients are as follows*:

$$\begin{array}{c} f\left(x\right)=\frac{1}{15}\sin|x^{R}+x^{J}+x^{K}|+i\frac{1}{12}\sin^{2}\left(x^{K}\right)+j\frac{1}{18}\cos\left(x^{I}+x^{K}\right)+k\frac{1}{15}\left|x^{K}\right|, \end{array}$$

$$\begin{array}{c} g\left(x\right)=\frac{1}{9}\tanh x^{R}+i\frac{1}{12}\left(\right|x^{I}+1|-|x^{J}-1\left|\right)+j\frac{1}{14}\cos^{2}\left(x^{J}\right)+k\frac{1}{18}\tanh x^{K}, \end{array}$$

$$\begin{array}{c} h\left(x\right)=\frac{1}{12}\cos\left|x^{R}+x^{I}\right|+i\frac{1}{18}\sin^{2}\left(x^{J}\right)+j\frac{1}{15}\cos\left(x^{J}+x^{K}\right)+k\frac{1}{16}\tanh x^{K}, \end{array}$$

$$\begin{array}{c} w\left(x\right)=\frac{1}{13}\cos\left(x^{R}\right)+i\frac{1}{14}\sin\left(x^{J}+x^{K}\right)+j\frac{1}{18}\cos^{2}\left(x^{J}\right)+\frac{1}{19}\sin\left(x^{I}+x^{K}\right), \end{array}$$

$$\begin{array}{c} a_{11}\left(t\right)=3+\cos t,\;\;a_{12}\left(t\right)=2+\sin\sqrt{2}t,\;\;a_{21}\left(t\right)=2+\sin t,\;\;a_{22}\left(t\right)=2+\left|\cos t\right|, \end{array}$$

$$\begin{array}{c} d_{11}\left(t\right)=\sin t+2,\;\;d_{12}\left(t\right)=2\cos\sqrt{3}t+4,\;\;d_{21}\left(t\right)=\sin\sqrt{2}t+4,\;\;d_{22}\left(t\right)=\sin t+5, \end{array}$$

$$\begin{array}{c} B_{11}\left(t\right)=0.02\cos\sqrt{2}t+0.01,\;\;B_{12}\left(t\right)=0.02\sin t,\;\;B_{21}\left(t\right)=0.03\sin\frac{\sqrt{3}}{3}t, \end{array}$$

$$\begin{array}{c} B_{22}\left(t\right)=0.02\cos t+0.01,\;\;C_{11}\left(t\right)=0.01\cos\frac{\sqrt{2}}{2}t,\;\;C_{12}\left(t\right)=0.01\sin t+0.02, \end{array}$$

$$\begin{array}{c} C_{21}\left(t\right)=0.03\sin\sqrt{3}t,\;\;C_{22}\left(t\right)=0.01\left|\cos t\right|,\;\;D_{11}\left(t\right)=0.06\cos\sqrt{2}t-0.01, \end{array}$$

$$\begin{array}{c} D_{12}\left(t\right)=0.01\sin t,\;\;D_{21}\left(t\right)=0.02\cos t,\;\;D_{22}\left(t\right)=0.02\cos\sqrt{5}t,\;\;E_{11}\left(t\right)=0.02\cos3t, \end{array}$$

$$\begin{array}{c} E_{12}\left(t\right)=0.03\cos\sqrt{2}t,E_{21}\left(t\right)=0.04\sin2t,\;\;E_{22}\left(t\right)=0.01\sin\sqrt{5}t+0.01, \end{array}$$

$$\begin{array}{c} T_{pq}\left(t\right)=0.2\sin\left(\sqrt{2}t\right)+i0.1\left|\cos t\right|+j0.3\sin t+k0.5\cos\sqrt{6}t. \end{array}$$

*We have*

$$\begin{array}{c} M_{f}^{R}=\frac{1}{15},\;\;M_{f}^{I}=\frac{1}{12},\;\;M_{f}^{J}=\frac{1}{18},\;\;M_{f}^{K}=\frac{1}{15},\;\;L_{f}^{R}=\frac{1}{15},\;\;L_{f}^{I}=\frac{1}{18}, \end{array}$$

$$\begin{array}{c} L_{f}^{J}=\frac{1}{15},\;\;L_{f}^{K}=\frac{1}{12},\;\;M_{g}^{R}=\frac{1}{9},\;\;M_{g}^{I}=\frac{1}{12},\;\;M_{g}^{J}=\frac{1}{14},\;\;M_{g}^{K}=\frac{1}{18}, \end{array}$$

$$\begin{array}{c} L_{g}^{R}=\frac{1}{9},\;\;L_{g}^{I}=\frac{1}{12},\;\;L_{g}^{J}=\frac{1}{12},\;\;L_{g}^{K}=\frac{1}{18},\;\;M_{h}^{R}=\frac{1}{12},\;\;M_{h}^{I}=\frac{1}{18}, \end{array}$$

$$\begin{array}{c} M_{h}^{J}=\frac{1}{15},\;\;M_{h}^{K}=\frac{1}{16},\;\;L_{h}^{R}=\frac{1}{12},\;\;L_{h}^{I}=\frac{1}{12},\;\;L_{h}^{J}=\frac{1}{15},\;\;L_{h}^{K}=\frac{1}{15}, \end{array}$$

$$\begin{array}{c} M_{w}^{R}=\frac{1}{13},M_{w}^{I}=\frac{1}{14},M_{w}^{J}=\frac{1}{18},M_{w}^{K}=\frac{1}{19},L_{w}^{R}=\frac{1}{13},L_{w}^{I}=\frac{1}{19},L_{w}^{J}=\frac{1}{14},L_{w}^{K}=\frac{1}{14}, \end{array}$$

$$\begin{array}{c} \sum_{C_{kl}\in N_{1}\left(1,1\right)}{\bar{B}}_{11}^{kl}=\sum_{C_{kl}\in N_{1}\left(1,2\right)}{\bar{B}}_{12}^{kl}=\sum_{C_{kl}\in N_{1}\left(2,1\right)}{\bar{B}}_{21}^{kl}=\sum_{C_{kl}\in N_{1}\left(2,2\right)}{\bar{B}}_{22}^{kl}=0.11, \end{array}$$

$$\begin{array}{c} \sum_{C_{kl}\in N_{1}\left(1,1\right)}{\bar{C}}_{11}^{kl}=\sum_{C_{kl}\in N_{1}\left(1,2\right)}{\bar{C}}_{12}^{kl}=\sum_{C_{kl}\in N_{1}\left(2,1\right)}{\bar{C}}_{21}^{kl}=\sum_{C_{kl}\in N_{1}\left(2,2\right)}{\bar{C}}_{22}^{kl}=0.08, \end{array}$$

$$\begin{array}{c} \sum_{C_{kl}\in N_{1}\left(1,1\right)}{\bar{D}}_{11}^{kl}=\sum_{C_{kl}\in N_{1}\left(1,2\right)}{\bar{D}}_{12}^{kl}=\sum_{C_{kl}\in N_{1}\left(2,1\right)}{\bar{D}}_{21}^{kl}=\sum_{C_{kl}\in N_{1}\left(2,2\right)}{\bar{D}}_{22}^{kl}=0.1, \end{array}$$

$$\begin{array}{c} \sum_{C_{kl}\in N_{1}\left(1,1\right)}{\bar{E}}_{11}^{kl}=\sum_{C_{kl}\in N_{1}\left(1,2\right)}{\bar{E}}_{12}^{kl}=\sum_{C_{kl}\in N_{1}\left(2,1\right)}{\bar{E}}_{21}^{kl}=\sum_{C_{kl}\in N_{1}\left(2,2\right)}{\bar{E}}_{22}^{kl}=0.11, \end{array}$$

$$\begin{array}{c} \bar{\tau }=\frac{1}{2},\bar{\delta }=\frac{1}{3},1-\alpha =\frac{1}{2},1-\beta =\frac{2}{3}. \end{array}$$

*Thus*, (*A*_1_)-(*A*_3_) *hold. Setting* *κ* = 2, *for* *p*, *q* = 1, 2, *we have*

$$\begin{array}{c} \vartheta_{pq}\approx 0.0691,\mu_{pq}\approx 0.6419,\vartheta \approx 0.4382<\kappa ,\mu \approx 0.6419<1, \end{array}$$

*which implies that* (*A*_4_) *is satisfied. Therefore, the drive system* (18) *has a unique almost periodic solution. Moreover, take* λ = 1, *we have*

$$\begin{array}{c} \gamma_{11}\approx -0.218,\gamma_{12}\approx -0.218,\gamma_{21}\approx -1.218,\gamma_{22}\approx -3.218,\gamma \approx -0.218<0. \end{array}$$

**Fig 1:**
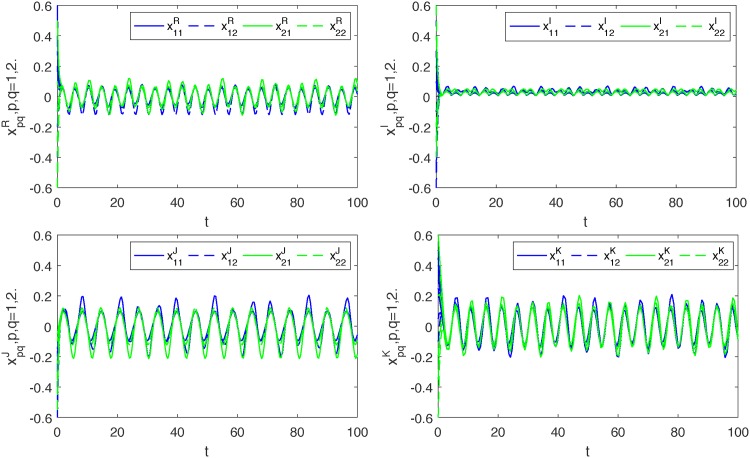
Transient states of four parts of *x*_*pq*_, *p*, *q* = 1, 2.

**Fig 2:**
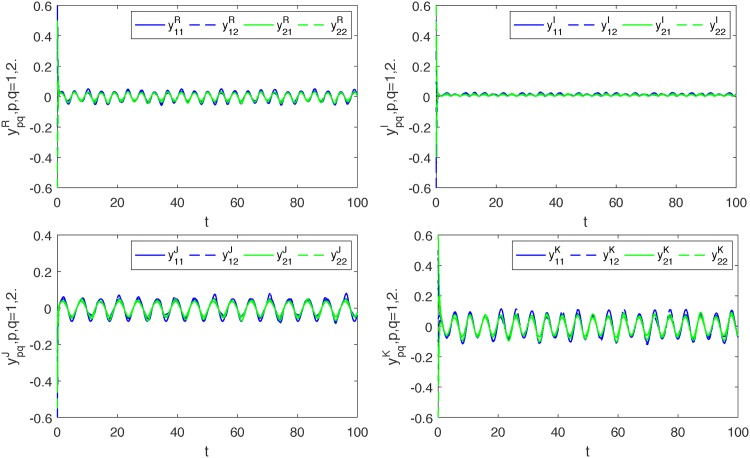
Transient states of four parts of *y*_*pq*_, *p*, *q* = 1, 2.

**Fig 3:**
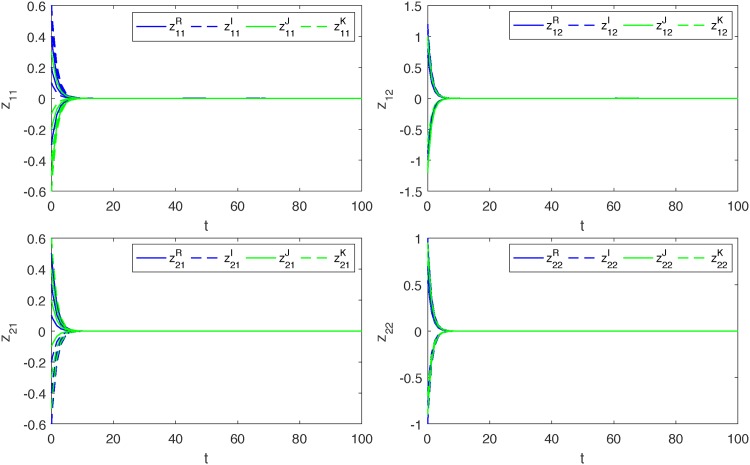
Synchronization.

**Fig 4:**
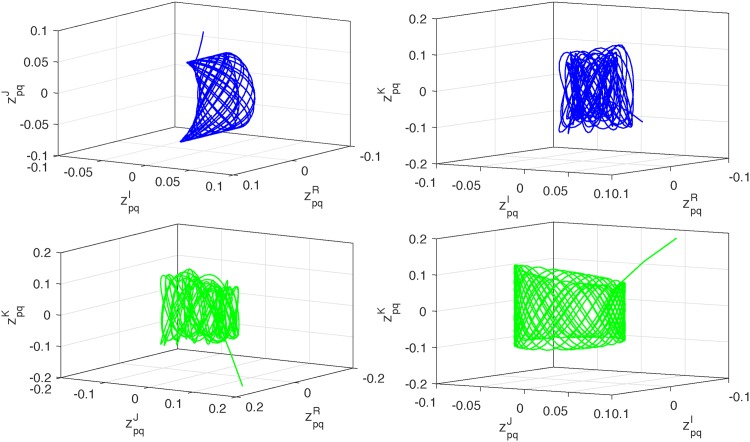
Curves of $z_{pq}^{\nu }$ (*p*, *q* = 1, 2, *ν* ∈ Λ) in 3-dimensional space for synchronization case.

*Thus*, (*A*_5_) *is also satisfied. Therefore*, (18) *and* (19) *are globally exponentially synchronized* (*see Figs* 1–4).

## Conclusion

In this paper, a class of QVSICNNs with mixed delays is studied. To the best of our knowledge, this is the first on studying the problem. Since QVSICNNs include RVSICNNs and CVSICNNs as special cases, our method of this paper can be applied to study the almost periodic synchronization problem of other types of neural networks including RVNNs and CVNNs.

In this paper, the almost periodic synchronization of a class of QVSICNNs with mixed delays is studied. To the best of our knowledge, this is the first on studying the problem. Since QVSICNNs include RVSICNNs and CVSICNNs as special cases, our method of this paper can be applied to study the almost periodic synchronization problem of other types of neural networks including RVNNs and CVNNs.
